# Holistic energy landscape management in 2D/3D heterojunction via molecular engineering for efficient perovskite solar cells

**DOI:** 10.1126/sciadv.adg0032

**Published:** 2023-06-07

**Authors:** Ke Ma, Jiaonan Sun, Harindi R. Atapattu, Bryon W. Larson, Hanjun Yang, Dewei Sun, Ke Chen, Kang Wang, Yoonho Lee, Yuanhao Tang, Anika Bhoopalam, Libai Huang, Kenneth R. Graham, Jianguo Mei, Letian Dou

**Affiliations:** ^1^ Davidson School of Chemical Engineering, Purdue University, West Lafayette, IN 47907, USA.; ^2^ Department of Chemistry, University of Kentucky, Lexington, KY 40506, USA.; ^3^ Chemistry and Nanoscience Center, National Renewable Energy Laboratory, Golden, CO 80401, USA.; ^4^ Department of Chemistry, Purdue University, West Lafayette, IN 47907, USA.; ^5^ Birck Nanotechnology Center, Purdue University, West Lafayette, IN 47907, USA.

## Abstract

Constructing two-dimensional (2D) perovskite atop of 3D with energy landscape management is still a challenge in perovskite photovoltaics. Here, we report a strategy through designing a series of π-conjugated organic cations to construct stable 2D perovskites and to realize delicate energy level tunability at 2D/3D heterojunctions. As a result, the hole transfer energy barriers can be reduced both at heterojunctions and within 2D structures, and the preferable work function shift reduces charge accumulation at interface. Leveraging these insights and also benefitted from the superior interface contact between conjugated cations and poly(triarylamine) (PTAA) hole transporting layer, a solar cell with power conversion efficiency of 24.6% has been achieved, which is the highest among PTAA-based n-i-p devices to the best of our knowledge. The devices exhibit greatly enhanced stability and reproducibility. This approach is generic to several hole transporting materials, offering opportunities to realize high efficiency without using the unstable Spiro-OMeTAD.

## Teaser

Conjugated ligand design for 2D/3D heterojunctions improves band alignment and charge transport in perovskite solar cells.

## INTRODUCTION

Perovskite solar cells (PSCs) have emerged as a strong candidate for future photovoltaic technology (*1*–*4*). However, achieving high efficiency together with long-term stability is still a challenge (*5*–*7*). More recently, the strategy of constructing two-dimensional/three-dimensional (2D/3D) heterostructures to passivate interface defects and improve stability has contributed to some of high-efficiency devices, including the record-performing PSCs (*8*–*14*). These studies have also revealed that energy level alignment at the 2D/3D heterojunction is crucial for efficient charge transfer (*15*, *16*). However, the methods of managing energy level alignment are limited to phase control of 2D perovskites (i.e., the thickness of the inorganic layers), owing to the insignificant contribution of conventional organic ligands to the density of states at the band edges in 2D perovskites (*17*).

**Fig. 1.**
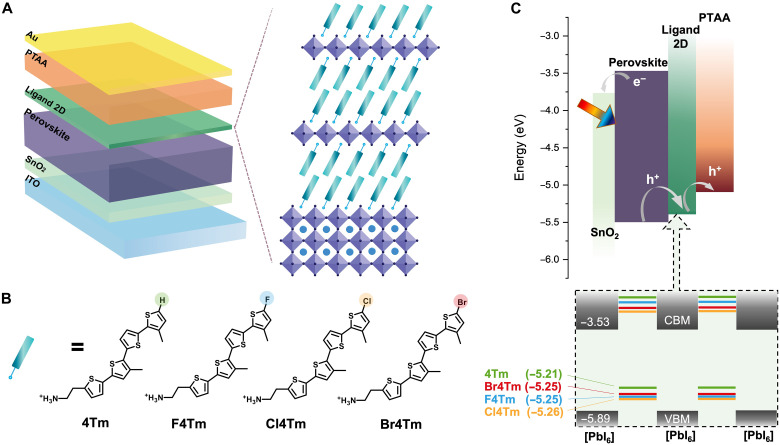
Device structure and conjugated ligands design. (**A**) Schematic of device structure used in this work with a 2D perovskite layer atop of 3D perovskite. (**B**) Chemical structures of the designed conjugated molecules in the form of ammonium cations. (**C**) The schematic of band alignment of device treated with conjugated ligands. The bottom shows the band alignments within the 2D perovskite structures formed with different conjugated ligands. The energy diagram is plotted as a schematic illustration, while the accurate energy levels are labeled in the plot. CBM, conduction band minimum; ITO, indium tin oxide.

Here, we developed a series of bulky semiconducting ligands with tunable energy levels that can form stable 2D perovskites (Fig. 1A). These π-conjugated ligands provide an opportunity for manipulating energy level alignment at 2D/3D heterojunctions through rational design of organic cations, thereby allowing more efficient hole extraction and reducing interface charge accumulation (*18*). Furthermore, the reduced energy level offset between the valence band maximum (VBM) of inorganic sheets and the highest occupied molecular orbital (HOMO) of conjugated organic layers in 2D perovskites, as well as preferable ligand packing geometry, allows more efficient hole transfer through the out-of-plane direction (*19*, *20*). This holistic energy landscape management of 2D/3D heterojunction in PSCs, together with the improved interface quality between perovskite and polymeric hole transporting layer (HTL) via π-conjugated ligands, enabled the demonstration of a remarkable power conversion efficiency (PCE) of 24.6% for poly(triarylamine) (PTAA)–based devices with n-i-p structure. The fine-tuned conjugated ligands allow more stable 2D structures and suppress interlayer ion migration, which result in improved photostability and thermal stability.

## RESULTS

### 2D perovskite structures and properties

Our general design strategy of the ligands is tethering an ammonium anchoring group to one end of a conjugated quaterthiophene unit (*18*, *21*) and having halogen substitution on the opposite end thiophene to manipulate the HOMO levels of the molecules (*22*). The chemical structures of the synthesized organic ligands feature different substituting groups, namely, F4Tm, Cl4Tm, and Br4Tm, all of which are derived from 4Tm (Fig. 1B). The synthesis of halogen-4Tm was based on electrophilic halogenation reactions to add functional groups and Stille coupling reaction to connect all the thiophene moieties together (details of synthesis in the Supplementary Materials). All ligands were converted to iodide salts through reactions with hydroiodic acid. The halogen substituent acts as an electron-withdrawing group that deepens the HOMO level of the ligands, from −5.21 eV for 4Tm to −5.25, −5.26, and −5.25 eV for F4Tm, Cl4Tm, and B4Tm ligands, respectively (fig. S1 and table S1).

Within the 2D perovskite structures formed with these ligands, type II band alignments are formed, because of the small bandgaps and shallow HOMO levels of the conjugated ligands (Fig. 1C). The type II alignment results in quenched photoluminescence observed from these 2D perovskite thin films (fig. S2). Unlike the conventional wide-bandgap ligands that usually form type I artificial quantum wells in 2D perovskites and generate energy barriers for out-of-plane charge transfer, the shallow HOMO levels of conjugated ligands reduce the energy barriers for hole transfer. However, the shallow HOMO level of 4Tm creates a reverse energy level offset with inorganic layers in 2D perovskites, which, in turn, can potentially trap holes. The halogen-4Tm reduces the energy level offsets, suggesting minimal energy barriers and a decreased probability of hole trapping, which, in principle, could facilitate out-of-plane hole transport within 2D perovskite structures.

**Fig. 2.**
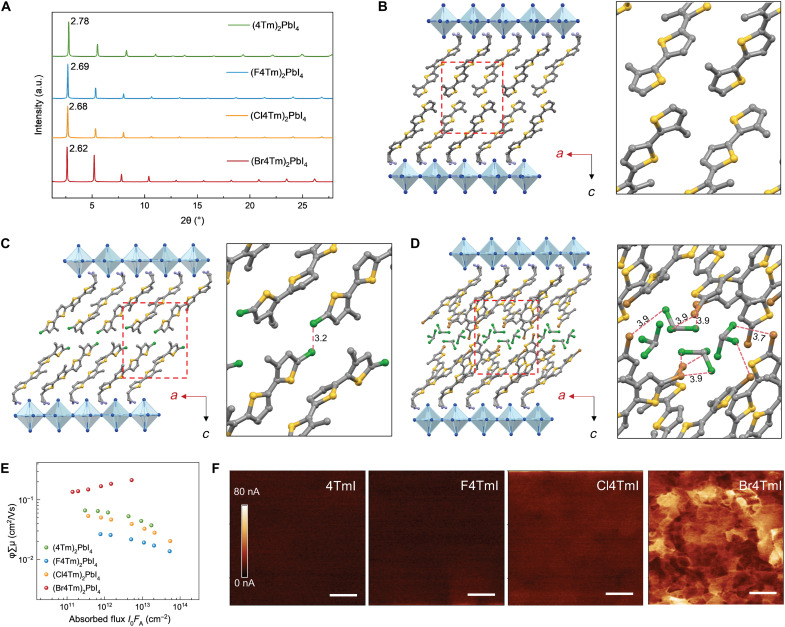
Structure and conductivity of 2D perovskites. (**A**) XRD of 2D perovskite (*n* = 1) thin films. (**B** to **D**) Single-crystal structures of 2D perovskites with different ligands. (B) (4Tm)_2_PbI_4_. (C) (Cl4Tm)_2_PbI_4_. (D) (Br4Tm)_2_PbI_4_. The right panels of (B), (C) and (D) present the zoom-in images of ligand interactions. (**E**) TRMC comparison of out-of-plane charge transport in *n* = 1 2D perovskite thin films. ϕ is the carrier generation yield and μ is the mobility for carriers. (**F**) Conductive atomic force microscopy (cAFM) of 2D perovskite thin films formed with different ligands. Scale bars, 1 μm. a.u., arbitrary units.

Besides the energy level alignment, a strong dependence between crystal structures and electronic properties also exists in 2D perovskites (*23*, *24*). Therefore, we characterized 2D perovskites through thin-film x-ray diffraction (XRD) and single-crystal analysis to gain insights from their crystal structures. The 2D perovskite thin films can be obtained by one-step spin coating followed by thermal annealing. The XRD patterns with characteristic planes—(001), (002), (003), etc.—are different from the XRD patterns of the aggregated ligands (fig. S3), confirming the formation of layered 2D structures (Fig. 2A). The absorption spectra exhibited distinctive exitonic peaks at around 512 nm, further supporting the formation of *n* = 1 2D Ruddlesden-Popper phase perovskites (fig. S4). The corresponding d-spacings of (4Tm)_2_PbI_4_, (F4Tm)_2_PbI_4_, (Cl4Tm)_2_PbI_4_, and (Br4Tm)_2_PbI_4_ were calculated as 3.18, 3.28, 3.28, and 3.37 nm from the 2θ of (001) planes at 2.78°, 2.69°, 2.68°, and 2.62°, respectively, which are correlated with the increased atomic radius of H, F, Cl, and Br. Note that, although fluorine has a smaller atomic radius than chlorine, (F4Tm)_2_PbI_4_ and (Cl4Tm)_2_PbI_4_ share similar interlayer distances, which is likely due to the interplay between atomic versus electrostatic attractive and repulsive forces.

To gauge the effect of molecular configuration on intermolecular packing, we further examined the single crystals of 2D perovskites formed with different ligands (Fig. 2, B to D). Single crystals of (Cl4Tm)_2_PbI_4_ were obtained by slow-cooling method, while solvent diffusion method was applied to grow crystals of (Br4Tm)_2_PbI_4_ (details in the Supplementary Materials). For F4TmI, suitable crystal specimens for accurate single-crystal structure determination proved challenging and resulted in high R-factors, although several attempts have been made, which is probably due to the weak interactions between F4Tm layers (fig. S5). The average in-plane Pb─I─Pb bond angle is 151.6° and the Pb─I bond length is 3.17 Å (horizontal) in (Cl4Tm)_2_PbI_4_. We observed a small distance of 3.2 Å between alternating Cl─Cl atoms within two Cl4Tm layers, which indicates the existence of weak halogen interaction (considering the 1.75-Å van der Waals radius of a single chlorine atom) and could contribute to the stabilization of the structure. The (Br4Tm)_2_PbI_4_ single-crystal structure was resolved with chloroform solvent molecules trapped between the ligand layers, from which Br─Cl halogen interaction between Br4Tm and chloroform were observed because of the shortened interatom distance. Because of solvent intercalation, the inorganic lattice of (Br4Tm)_2_PbI_4_ has less distortion compared with (Cl4Tm)_2_PbI_4_, with the average in-plane Pb─I─Pb bond angle as 152.6° and Pb─I bond length as 3.14 Å (horizontal). However, part of the conjugated ligand molecules in (Br4Tm)_2_PbI_4_ crystal structure exhibit more distortion compared to (Cl4Tm)_2_PbI_4_, which could increase the formation barrier of this 2D structure (*25*).

To understand the relationship between crystal structures and the electronic properties, we applied time-resolved microwave conductivity (TRMC) measurements to characterize the out-of-plane carrier transport in 2D perovskite thin films (Fig. 2E) (*13*, *26*). The (Br4Tm)_2_PbI_4_ sample has the highest yield-mobility product, which is a factor of 2 to 4 larger than that of (Cl4Tm)_2_PbI_4_ and (4Tm)_2_PbI_4_. However, the (F4Tm)_2_PbI_4_ sample exhibits obviously lower mobility, which could be correlated with the difficulty of achieving high-quality (F4Tm)_2_PbI_4_ single crystal and the repulsion between fluorine atoms. We also performed conductive atomic force microscopy (cAFM) to examine the spatial distribution of the current path in these thin films (Fig. 2F). In agreement with the TRMC results, the (F4Tm)_2_PbI_4_ sample exhibits the lowest conductivity, while the (Br4Tm)_2_PbI_4_ sample preserves the highest conductivity. The higher out-of-plane conductivity of the (Br4Tm)_2_PbI_4_ thin film than the Cl4TmI sample has also been confirmed with single-crystal conductivity (fig. S6). The single-crystal structures show that the outer thiophenes in (Cl4Tm)_2_PbI_4_ crystals favor in-plane edge-to-edge arrangement between the neighboring Cl4Tm layers, similar to 4Tm samples, which may not support efficient charge transfer (fig. S7). In contrast, the (Br4Tm)_2_PbI_4_ crystal exhibits face-to-face ligand packing geometry, thus exhibiting higher out-of-plane conductivity (*23*, *25*, *27*, *28*). However, the cAFM images reveal that (Br4Tm)_2_PbI_4_ thin film, although having higher conductivity, has greater heterogeneity in the current path distribution, which may result from the nonuniform crystallinity and the potential higher formation energy of this 2D structure.

### 2D/3D heterostructure formation and characterization

**Fig. 3.**
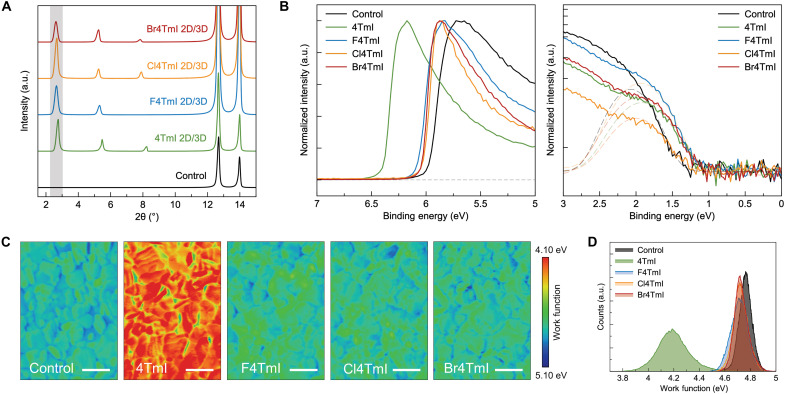
Formation of 2D/3D heterojunction and surface energetic characterization. (**A**) XRD patterns of 3D perovskite thin films without and with surface treatment. (**B**) Ultraviolet photoelectron spectroscopy (UPS) results of various surface treatments. Left: Secondary cut-off region; right: on-set region (light source of 10.2 eV). (**C**) Kelvin probe force microscopy (KPFM) surface potential maps of perovskite thin films with different surface treatments. Scale bars, 2 μm. (**D**) Work function distribution extracted from KPFM results.

We then investigated the formation of 2D/3D heterostructures on the surface of 3D perovskites for photovoltaic devices. The 2D structures were formed by means of coating the corresponding ligand solution on the surface of 3D perovskite, followed by thermal annealing. To enhance the 2D perovskite signal for XRD characterization, we extended the reaction time between 3D perovskite and the ligands by dropping the ligand solutions and waiting for 60 s before spinning. The thin-film XRD suggests that *n* = 1 2D structures form with all the investigated ligands, as evidenced by the diffraction peaks at low angles (<10°) (Fig. 3A), which match with the peaks from pure 2D perovskite thin films. The minor shift of the low-angle peaks from pure 2D perovskites to the 2D/3D heterostructures may be induced by the slight lattice distortion caused by 2D/3D interfacial strain. Only the *n* = 1 phase is formed on the surface with horizontal orientation, while high-*n* number phases are absent for all ligands investigated. We ascribe the stability of 2D structures, the rigidity of the ligands, and their unlikely penetration into the 3D structure due to steric bulk, all as factors that prevent formation of other phases during the treatment. The exclusive formation of *n* = 1 phase in 2D/3D junctions distinguishes these ligands from conventional small ligands and eliminates the influence of phase impurity in the surface characterizations. The AFM images indicate the unchanged surface morphology of perovskite with short-time ligand treatment (fig. S8). The x-ray photoelectron spectroscopy (XPS) conducted on ligand-treated 3D perovskite thin films demonstrates the existence of oligothiophene ligands on the 3D perovskite surface from the peaks of S 2p at 164 eV (fig. S9). Moreover, the characteristic peaks of F 1s (687 eV), Cl 2p (202 eV), and Br 3d (71 eV) show up in the XPS results of F4TmI, Cl4TmI, and Br4TmI thin films, respectively, which belong to the terminal substituents of the corresponding ligands.

We proposed to modulate the surface potential and 2D/3D band alignment via rational design of ligand molecular configurations. Through ultraviolet photoelectron spectroscopy (UPS) measurements (Fig. 3B), we verified that ligand treatment upshifts the VBM in comparing with the control film (untreated), based on the aligned Fermi level, which is induced by the shallower HOMO levels of the conjugated ligands (fig. S10 and table S2). The upshifted VBM generates a more p-type surface, which is expected to facilitate efficient hole transfer from perovskite to PTAA (with a reported HOMO at −5.2 eV) (*21*). The shallow HOMO levels of these conjugated ligands also allow them to directly extract holes from 3D perovskites, which is verified by time-resolved photoluminescence (TRPL) measurement with a biexponential decay and fast hole extraction process within 20 ns (fig. S11).

The work functions (WFs) have also been extracted from UPS measurements, which show notable differences between 4TmI and halogen-4TmI treatment. The 4TmI treatment notably reduces WF to 3.82 eV from the 4.22 eV of the control film. In contrast, the reduction of WF is smaller when halogen substitutions are introduced, showing less than 0.1-eV changes (Fig. 3B and table S2). Kelvin probe force microscopy (KPFM) measurements revealed WF distribution differences at the ligand-treated surfaces (Fig. 3C). The average WF of 4TmI-treated surface reduced to 4.18 eV from 4.76 eV for the control film, while the change of WF of the halogen-4TmI–treated surface was negligible (Fig. 3D), which is in accordance with the trend observed in UPS analysis.

The band alignment formed with 4TmI surface treatment showed that the perovskite surface has shallower VBM compared with the bulk material, but a negative ΔWF could generate a potential well at the interface of the 2D/3D heterostructure and trap the electrons (fig. S11). Unlike the downshifted VBM of other 2D/3D heterostructures formed with wide-bandgap ligands, the hole transfer benefits from the upshifted VBM of perovskite surface with conjugated ligands. However, the trapped and accumulated electrons at perovskite/HTL interface can cause charge trapping–induced defects (*4*, *29*). In comparison, the 2D/3D heterostructures formed with halogen-4TmI cause minimum WF shift, thus being unlikely to create a deep potential well at interfaces. The reduced charge-trapping defects in halogen-4TmI–treated films are evidenced by the enhanced photoluminescence (PL) intensities with a film structure of glass/3D perovskite/2D perovskite (fig. S12). The reduced bimolecular recombination was further affirmed with intensity-dependent photoconductivity transient characterization from TRMC measurements (fig. S13).

In addition, benefitted from the exceptional out-of-plane transport of the *n* = 1 2D perovskite structures containing Cl4TmI and Br4TmI, the coverage of 2D perovskite atop of 3D thin films does not decrease the carrier mobility (fig. S14), which further indicates that the enhanced out-of-plane charge transport in 2D perovskites is critical to reduce charge transfer barriers at the 2D/3D heterojunction. The cAFM results even suggest slightly higher conductivity in Cl4TmI-treated films than that in Br4TmI samples (fig. S15), which we attribute to the different formation energy of 2D/3D heterojunction with different ligands.

### Carrier dynamics and device performance

**Fig. 4.**
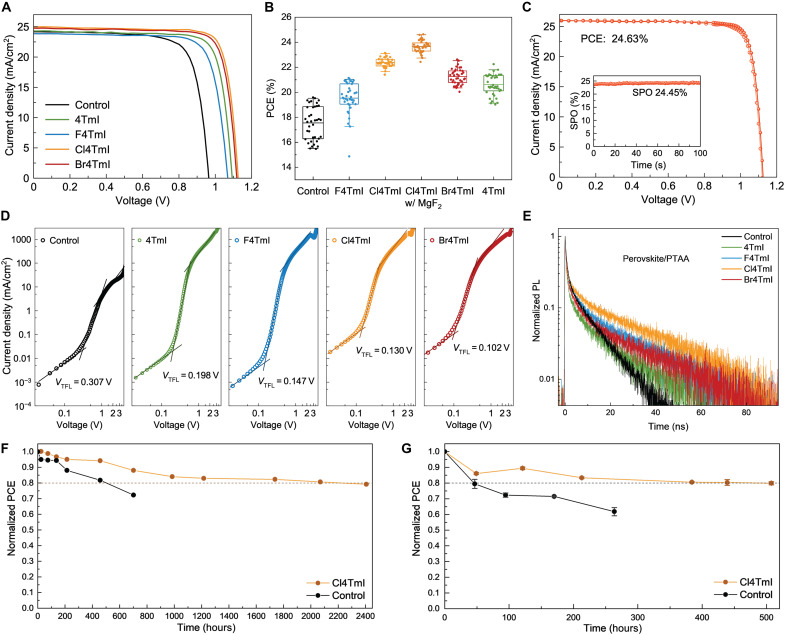
Device performance and carrier dynamics. (**A**) *J-V* characteristics of PSCs based on different surface treatments. (**B**) Statistics showing the PCE distribution of the devices with different treatments. (**C**) *J-V* characteristic of the champion device with reverse (solid dot) and forward (open dot) scans. Inset: SPO (stabilized power output) of the corresponding device. (**D**) Space-charge–limited current (SCLC) characteristics of the hole-only devices treated with different ligands. (**E**) TRPL spectra of the glass/perovskite/PTAA films with different ligand treatments between perovskite and PTAA. (**F**) Long-term stability of PSCs tested at 65°C under N_2_ environment. The initial PCE of Cl4Tm and control devices are 20.9 and 17.5%, respectively. (**G**) Photostability tracking under the open-circuit condition of PSCs without and with Cl4TmI treatment. The measurement was performed in the N_2_-filled glovebox with encapsulation. The initial PCE of Cl4TmI and control devices are 21.3 and 18.3%, respectively.

We investigated the impact of these 2D/3D heterostructures on the photovoltaic performance by fabricating the PSCs using an n-i-p device architecture, ITO/SnO_2_/perovskite/PTAA/Au, with different surface ligand treatments. In agreement with our analysis, the Cl4TmI and Br4TmI surface treatment results in remarkably enhanced PCE, which outperformed the devices treated with 4TmI and F4TmI. A typical set of *J-V* curves of devices fabricated with different ligands is shown in Fig. 4A. The nonideal energy alignment of 4TmI and the unstable 2D crystal structure and low conductivity of (F4Tm)_2_PbI_4_ are the limiting factors of these two ligands. Comparing with Cl4TmI, slightly less improvement with Br4TmI treatment was achieved, which could correspond to the different conductivity of the 2D/3D heterostructure formed with Cl4TmI and Br4TmI, as well as the heterogeneity of conductivity distribution in (Br4Tm)_2_PbI_4_ thin film. To verify the reproducibility of the results, we provide the statistics for at least 38 devices of each condition (Fig. 4B and fig. S16). Furthermore, we compared our conjugated ligands to the devices passivated with butylammonium iodide and phenethylammonium iodide (fig. S17). Neither of these commonly used ligands was able to produce comparable PCEs as Cl4TmI and Br4TmI, which suggests the critical role of the semiconducting ligands in 2D/3D heterostructures. In addition to the FA_0.9_MA_0.05_Cs_0.05_PbI_3_ perovskite composition, we also investigated the effect of Cl4TmI surface treatment on PSCs with a composition of double halide (FA_0.88_MA_0.07_Cs_0.05_PbI_2.89_Br_0.11_) and also found an increase in PCE (fig. S18).

Note that we focused on PTAA doped with a hydrophobic Lewis acid as HTL, because it is considered as a more stable hole transporting material than Spiro-OMeTAD and does not need lithium salt for efficient doping, which eliminates a degrading factor of FAPbI_3_. In addition, PTAA is known to be less sensitive to processing conditions with no air aging requirement and leads to devices with better reproducibility. However, the limiting factor of the application of PTAA is the interface quality with perovskite, leading to low PCE values in conventional devices (*30*). Here, the designed conjugated ligands increase the hydrophobicity of perovskite surface (fig. S19), forming perfect atomic registry to the perovskite lattice, and share similar aromaticity and improve the interface contact between perovskite and PTAA. This critical interface supports efficient hole transfer and results in increased open circuit voltage (*V*_OC_) and fill factor (*FF*). With further optimization of ligand concentrations and passivation procedures (figs. S20 to S23), and together with an antireflection layer coating, a champion device with a PCE of 24.63% was achieved through Cl4TmI treatment (*V*_OC_ = 1.125 V, *FF* = 84.32%, short circuit current density (*J*_SC_) = 25.96 mA/cm^2^) with small hysteresis comparing with control device (Fig. 4C and fig. S24), which is the highest reported PCE among PTAA-based PSCs to the best of our knowledge (*31*–*33*). A stabilized power output of 24.45% was also measured at maximum power point (Fig. 4C), and the external quantum efficiency (EQE) spectrum was confirmed with an integrated *J*_SC_ of 25.5 mA/cm^2^ (fig. S25). The reproducibility of PTAA-based devices with our strategy has also been demonstrated by showing the small deviation from person-to-person variations of four different researchers (fig. S26).

We further conducted space-charge–limited current (SCLC) to probe the hole mobilities in the hole-only devices (Fig. 4D). Devices treated with different conjugated ligands all exhibit almost two orders of magnitude increase in hole mobility compared with the control device, which highlights the critical role of interface treatment in improving the interface contact between perovskite and PTAA and enhancing charge transport efficiency (table S3) (*34*, *35*). Note that, although the F4TmI-treated perovskite thin film exhibits lower mobility than the control film by TRMC, the F4TmI device still shows higher hole mobility than the control device, further supporting the notion that the PTAA/perovskite interface is the major bottleneck at the device level. In addition, the trap densities extracted from the trap-filled limit voltage (*V*_TFL_) in SCLC plots indicate reduced defect density in ligand-treated devices (table S3) (*36*). TRPL measurements on samples with a structure of glass/perovskite/ligand/PTAA were used to assess the defect densities at interfaces and the charge extraction processes (Fig. 4E and table S4). Ligand-treated films, benefitting from the gradient energy level alignment, exhibit faster charge extraction, as indicated by the fast decay of PL at initial stage. In the second stage of the biexponential decay, the PL lifetime is dominated by defect-induced monomolecular recombination. The lifetime of Cl4TmI-treated perovskite is 20.65 ns, two times higher than the 9.34 ns of the control sample that is correlated with the decreased defect density and increased *V*_OC_ with surface passivation (*37*, *38*). The suppressed mobile ions in Cl4TmI-treated devices were further evidenced by the capacitance-frequency profiles of the device compared with the control device (fig. S27). The decreased capacitance at a low-frequency region (<100 Hz) in the Cl4TmI-treated device reveals less-mobile ion response, which is correlated with the suppressed defect density at the surface (*39*). The tuned energy level alignment of perovskite surface not only results in efficient charge transfer but also affects the built-in potential (*V*_bi_). The Mott-Schottky plot analysis of ligand-treated devices reveals higher *V*_bi_ compared with that of control device, which is induced by the proper band alignment and the efficient hole extraction, among which Cl4TmI device exhibits the highest *V*_bi_ (fig. S28 and table S5). In addition, the improved energy level alignment also reduces the energy barriers for charge injection when the device is operated as a light-emitting diode (LED) and results in an electroluminescence EQE of 4.6% (fig. S29). This strategy is generically applicable to multiple small molecular and polymeric HTL materials with remarkably improved performance (fig. S30), representing a critical design element for future PSC interfacial engineering.

Last, we tracked the thermal stability of the unencapsulated devices at 65°C under N_2_ environment (Fig. 4F). The device treated with Cl4TmI maintained 80% of its initial efficiency after 2220 hours of heating, while the PCE of the control device dropped by 20% within 640 hours, showing only one-third of *T*_80_ lifetime compared with the Cl4TmI-treated device. The improved thermal stability is benefitted from the inhibition of ion migration at interface with the 2D layer modification, characterized with time-of-flight secondary ion mass spectrometry (TOF-SIMS) (fig. S31). We also checked the photostability of the encapsulated device under continuous illumination at an open-circuit condition in N_2_ environment (Fig. 4G). The photogenerated charge carriers are unextractable under an open-circuit condition and can accumulate at interfaces, accelerating the degradation process. The Cl4TmI-treated device exhibits 20% relative efficiency drop after 507 hours, while the control device decreased to 80% of its initial efficiency within 50 hours. In addition, the Cl4TmI-treated device maintained 80% of its initial PCE after 496 hours of continuous operation under illumination using maximum power point tracking under N_2_ environment with temperature around 45°C (fig. S32).

## DISCUSSION

We present the pivotal role of organic molecular design in holistically manipulating the physical and energy landscapes to reduce charge transfer barriers and defects at 3D/2D/HTL heterointerfaces. The synergistic impact of tunable energy levels and molecular packing geometry of conjugated ligands in 2D structures facilitates efficient out-of-plane charge transport in 2D perovskites, while the manageable energy alignment at 2D/3D heterojunction supports charge extraction in device level. Our strategy of molecular engineering enabled us to demonstrate a remarkable PCE of 24.6% for n-i-p PSCs based on PTAA HTLs, addressing long-standing interfacial issues in PTAA-based devices.

## MATERIALS AND METHODS

### Materials

SnO_2_ (15% colloidal solution) was purchased from Alfa Aesar. PTAA was purchased from 1-Material. 4-Isopropyl-4′-methyldiphenyliodonium tetrakis(pentafluorophenyl)borate (TPFB) was purchased from TCI. PbI_2_ (99.9999% perovskite grade), CsI, KOH, polyethylenimine (80% ethoxylated solution) (PEIE), 2,5-bis(tributylstannyl)thiophene, 2-bromo-3-methylthiophene, tris(dibenzylideneacetone)dipalla-dium(0), tri(*o*-tolyl)phosphine, *n*-butyl lithium, *N*-fluorobenzenesulfonimide, tetrabutylammonium hexafluorophosphate (TBAPF_6_), lithium diisopropylamide, and tributyltin chloride were purchased from Sigma-Aldrich. Formamidinium iodide, methylammonium iodide, methylammonium chloride, and methylammonium bromide were purchased from Greatcell Solar. Chlorobenzene, dimethylformamide (DMF), dimethyl sulfoxide (DMSO), and iso-propanol (IPA) are all anhydrous and were purchased from Sigma-Aldrich.

### 2D perovskite single-crystal growth

The (Cl4Tm)_2_PbI_4_ single crystal was obtained by slow cooling method. Two milligrams of Cl4TmI, 5 mg of PbI_2_, 200 μl of 57 wt% (weight %) of hydroiodic acid (HI) solution, and 100 μl of H_3_PO_2_ were added to 1 ml of ethanol. The mixture was heated at 100°C until dissolved and then the solution was slowly cooled to room temperature over the course of 60 hours. By filtration and ethanol wash, orange needle–like single crystals were collected.

The (Br4Tm)_2_PbI_4_ single crystal was obtained by solvent diffusion method. A total of 0.01 M Br4TmI and 0.005 M PbI_2_ were dissolved in gamma-butyrolactone at 70°C overnight. After dissolution, 0.1 ml of the precursor solution in a small vial was placed in a large vial with 3 ml of chloroform as antisolvent. Orange plate–like crystals precipitated out after 3 days. The crystallization process was conducted at room temperature in atmosphere.

### 2D perovskite film fabrication

Glass slides were cleaned using soap water, water, acetone, and isopropanol for 15 min in ultrasonic bath; dried with a nitrogen gun; and then were used as substrates for 2D perovskite thin-film fabrication. The clean substrates were treated with UV Ozone cleaner for 20 min before use. The precursor solution (200 μl) for spin-coating was prepared by dissolving 0.2 M ligands and 0.1 M PbI_2_ in dry DMF/DMSO (in 4/1 ratio) at 70°C. For spin-coating, 20 μl of precursor solution was used with a spin speed at 2000 rpm for 30 s. In the end, as-prepared thin films were transfer to a heating plate to anneal at 100° to 200°C for 10 min. The above precursor solution preparation, spin,-coating and thermal annealing processes were conducted in a N_2_ glove box.

### PSC fabrication

The ITO/glass substrates were cleaned extensively with deionized water, acetone, and isopropanol. The cleaned substrates were UVO treated for 30 min before using. SnO_2_ (15 wt%) stock solution was diluted with IPA:H_2_O (v/v = 1:1) to 2.14%, and then 0.61% polyethylenimine ethoxylated (PEIE) solution was added to the SnO_2_ solution. The SnO_2_ solution was spun-coated onto the ITO substrate at 3000 rpm, followed by annealing at 150°C for 30 min. After UVO treating the SnO_2_ surface for 10 min, 10 mM KOH solution was coated on to the substrate at 3000 rpm, followed by another annealing at 150°C for 30 min. The SnO_2_ substrates were UVO treated for 10 min before transferred into the glovebox to conduct the following process. The perovskite film was coated on SnO_2_ substrate with a two-step method. In the first step, the PbI_2_ solution (691.5 mg of PbI_2_, 19.5 mg of CsI, 900 μl of DMF, and 100 μl of DMSO) was spun-coated onto the substrate at 1500 rpm for 30 s, followed by annealing at 70°C for 1 min. After cooling down, the cation solution (90 mg of FAI, 5 mg of MAI, 10.8 mg of MACl, and 1 ml of IPA) was coated on the PbI_2_ film at 1500 rpm for 30 s, followed by annealing at 150°C for 15 min under ambient environment (50 to 70% relative humidity). For FA_0.88_MA_0.07_Cs_0.05_PbI_2.89_Br_0.11_ devices, the composition of cation solution changed to 90 mg of FAI, 3.2 mg of MABr, and 10.8 mg of MACl in 1 ml of IPA. The perovskite film was then transferred back to an N_2_-filled glovebox for ligand and HTL coating. The conjugated ligands were dissolved in a mixture of chlorobenzene and isopropanol (9:1 v/v) with a concentration of 0.5 mg/ml. Because of the properties of oligothiophene ammonia iodide salt, a mixture of polar solvent and nonpolar solvent was selected for better solubility. The concentration of the ligand solution was optimized on the basis of its solubility and influence on device performance. For ligand-treated devices, the ligand solution was coated on perovskite surface at 4000 rpm, followed by annealing at 100°C for 2 min. PTAA HTL was prepared in chlorobenzene solution (30 mg/ml), doped with 11% TPFB. The doped PTAA solution was stirred at 45°C overnight to ensure full dissolving and doping of PTAA. TPFB, a hydrophobic Lewis acid, is selected here as the dopant to avoid the incorporation of small mobile Li^+^ into the system while maintaining the hole mobility of PTAA. PTAA solution was spun on perovskite film at 4000 rpm for 30 s, followed by annealing at 80°C for 5 min. Last, 90 nm of gold was evaporated onto the device with shadow mask to determine the device area. For champion devices, 105 nm of MgF_2_ was evaporated on to the glass substrate as an antireflection layer. Four pixels are made on each device substrates, and more than 10 batches of devices were fabricated to confirm the reproducibility.

### Chemical characterization

NMR spectra were collected using a Bruker AV-III-400-MHz spectrometer. The cyclic voltammetry was completed with a CHI660 electrochemical analyzer. The working electrode is a glassy carbon, and the counter electrode is a Pt wire. Ag/AgCl is the reference electrode, and the electrolyte is 0.1 M TBAPF_6_, 1 mM ligand in dry dichloromethane. The measurement was conducted at a scan rate of 40 mV/s under a N_2_-purged environment. Ferrocene was added to the electrolyte to calibrate the reference electrode.

### Thin film characterization

The 2D/3D thin films used for characterizations were prepared with the same methods used in device fabrication, unless otherwise noted. The thin-film XRD patterns were characterized with a Rigaku Smart Lab equipped with a Cu Kα source (λ = 1.54056 Å). The 2D layer formed on top of 3D thin films used for XRD characterization was prepared by allowing the ligand solution (0.5 mg/ml) to stay on the surface of 3D thin films for an extended time before spun off. Steady-state PL spectra were measured using a SpectraPro HRS-300. TRPL spectra were collected using a time-correlated single-photon counting apparatus (PicoQuant) with a picosecond pulsed laser at 447 nm as the excitation source. The water contact angle image was captured with Ramé-hart Model 200. Fractional absorptance was measured inside a Cary 6000i integrating sphere center-mounted sample holder to account for diffuse and specular scatter off the film and quartz substrate independently from absorption of photons by each film.

All TRMC measurements on the 3D/2D perovskite films were recorded at 650-nm excitation, over one order of magnitude in excitation power at the lowest fluence limit, while continuously purging the microwave cavity with dry nitrogen. For the *n* = 1 2D perovskite films, 512 nm was used as the excitation wavelength, with over two orders of magnitude in power. The pulse width of the 10-Hz excitation beam is approximately 5 ns, and photoconductivity transients were recorded over a 500-ns time window for all samples. Fractional absorptance was used to quantify the TRMC yield-mobility product data.

The UPS characterization was conducted with an H Lyman-α photon source (E-LUX 121), which emits photon energy of 10.2 eV, and the electrons were detected with a 5.85-eV pass energy using a multichannel plate detector and a hemispherical electron energy analyzer while the perovskite samples were biased at −5 V. The XPS measurements were performed with the same PHI 5600 ultrahigh vacuum system and used an Al Kα source (1486.6 eV; PHI 04-548 dual-anode x-ray source) for excitation.

Both the KPFM and cAFM images were obtained using the Asylum Research Cypher ES Environmental AFM in the air. The samples for KPFM and cAFM measurements were prepared on ITO/glass substrates as conductive substrates, and the AFM tips used during the measurement were Ti/Pt-coated AC240TM-R3 tips (Oxford Instruments). All the KPFM measurements were conducted with the same tip and repetitively checked with another tip. In addition, during each KPFM measurement, the perovskite sample was electrically grounded with a conductive sample holder, which allows the surface potential of all the samples having the same zero point. For cAFM, all the measurements were conducted in the dark. Both the 2D thin-film samples and 2D single-crystal samples were biased at 3 V, while the 3D perovskite thin films treated with different ligands were biased at 0.8 V. The 2D single-crystal samples were transferred on to the substrates through tape-peeling method.

Positive high–mass resolution depth profile was performed using a TOF-SIMS NCS instrument, which combines a TOF.SIMS5 instrument (ION-TOF GmbH, Münster, Germany) and an in situ scanning probe microscope (NanoScan, Switzerland) at the Shared Equipment Authority from Rice University.

### Device characterization

The *J*-*V* curve characteristics were performed with simulated AM1.5G irradiation (100 mW/cm^2^), produced by a Xenon lamp–based solar simulator (Enlitech, SS-F5-3A). The light intensity was calibrated with Si reference cell certified by National Renewable Energy Laboratory (NREL). The active area was defined by Au electrode and was measured under a microscope (around 0.05 cm^2^). The voltage scan was conducted under both reverse scan (1.2 to −0.1 V) and forward scan (−0.1 to 1.2 V). The voltage step is 40 mV from −0.1 to 0.8 V and 10 mV from 0.8 to 1.2 V. The devices were measured in a nitrogen-filled glovebox. The EQE results were collected at zero bias under an ambient environment on a homebuilt equipment using a preamplifier and a lock-in amplifier at a chopper frequency of around 161 Hz. The light source was calibrated with a reference Si (818-UV-L) diode. The SCLC characterization was performed with hole-only devices (ITO/PEDOT:PSS/perovskite/PTAA/Au), and the voltage was scanned from 0 to 5 V with step size of 20 mV under a dark condition. The hole mobilities were calculated by fitting the curve using Mott-Gurney law in Child’s regime with the following equation

$$J=\frac{9\varepsilon_{0}\varepsilon_{r}\mu V^{2}}{8L^{3}}$$

where ε_0_ is the vacuum permittivity, ε_r_ (i.e., 25) is the relative dielectric constant, μ is the charge mobility, *V* is the applied voltage, *L* is the thickness of perovskite (700 nm), and *J* is the current density.

The defect density was calculated on the basis of the following equation

$$\frac{N_{t}=2\varepsilon_{0}\varepsilon_{r}V_{\mathrm{TFL}}}{qL^{2}}$$

where *V*_TFL_ is the onset voltage of the trap filled limit region and *q* is the elemental charge.

The capacitance-frequency measurements were performed at a frequency range of 1000 kHz to 100 mHz using a VersaSTAT electrochemical workstation (Ametek). The devices were measured with zero direct voltage bias and a sinusoidal perturbation of 20 mV. The Mott-Schottky analysis was performed on the same equipment at fixed frequency (10 kHz) with a scan from 0 to 1.2 V.

### Device stability measurement

For maximum power point stability test, the unencapsulated devices were exposed under continuous LED light source under N_2_ environment. The devices were biased with a voltage corresponding to their maximum power point, and the current was continuously tracked. For open-circuit stability test, the encapsulated devices were exposed under continuous LED light source under N_2_ environment. All the stability tests were performed without temperature control, and the device temperature was around 45°C. For thermal stability test, the devices were located on a hot plate with designated temperature under N_2_ environment.

## Supplementary Materials

### This PDF file includes:

Supplementary Text Figs. S1 to S32 Tables S1 to S7 References

## References

[1] M. Grätzel, The rise of highly efficient and stable perovskite solar cells. Acc. Chem. Res. 50, 487–491 (2017). 28945408 10.1021/acs.accounts.6b00492

[2] H. Min, D. Y. Lee, J. Kim, G. Kim, K. S. Lee, J. Kim, M. J. Paik, Y. K. Kim, K. S. Kim, M. G. Kim, T. J. Shin, S. I. Seok, Perovskite solar cells with atomically coherent interlayers on SnO_2_ electrodes. Nature 598, 444–450 (2021). 34671136 10.1038/s41586-021-03964-8

[3] Y. Zhao, Z. Qu, S. Yu, T. Shen, H. Deng, X. Chu, X. Peng, Y. Yuan, X. Zhang, J. You, Inactive (PbI_2_)_2_RbCl stabilizes perovskite films for efficient solar cells. Science 377, 531–534 (2022). 35901131 10.1126/science.abp8873

[4] S. Tan, T. Huang, I. Yavuz, R. Wang, T. W. Yoon, M. Xu, Q. Xing, K. Park, D.-K. Lee, C.-H. Chen, R. Zheng, T. Yoon, Y. Zhao, H.-C. Wang, D. Meng, J. Xue, Y. J. Song, X. Pan, N.-G. Park, J.-W. Lee, Y. Yang, Stability-limiting heterointerfaces of perovskite photovoltaics. Nature 605, 268–273 (2022). 35292753 10.1038/s41586-022-04604-5

[5] Y. Wang, I. Ahmad, T. Leung, J. Lin, W. Chen, F. Liu, A. M. C. Ng, Y. Zhang, A. B. Djurišić, Encapsulation and stability testing of perovskite solar cells for real life applications. ACS Mater. Au. 2, 215–236 (2022). 36855381 10.1021/acsmaterialsau.1c00045 PMC9888620

[6] R. Wang, M. Mujahid, Y. Duan, Z. K. Wang, J. Xue, Y. Yang, A review of perovskites solar cell stability. Adv. Funct. Mater. 29, 1808843 (2019).

[7] Q. Jiang, J. Tong, Y. Xian, R. A. Kerner, S. P. Dunfield, C. Xiao, R. A. Scheidt, D. Kuciauskas, X. Wang, M. P. Hautzinger, R. Tirawat, M. C. Beard, D. P. Fenning, J. J. Berry, B. W. Larson, Y. Yan, K. Zhu, Surface reaction for efficient and stable inverted perovskite solar cells. Nature 611, 278–283 (2022). 36049505 10.1038/s41586-022-05268-x

[8] Q. Jiang, Y. Zhao, X. Zhang, X. Yang, Y. Chen, Z. Chu, Q. Ye, X. Li, Z. Yin, J. You, Surface passivation of perovskite film for efficient solar cells. Nat. Photonics 13, 460–466 (2019).

[9] Z. Wang, Q. Lin, F. P. Chmiel, N. Sakai, L. M. Herz, H. J. Snaith, Efficient ambient-air-stable solar cells with 2D–3D heterostructured butylammonium-caesium-formamidinium lead halide perovskites. Nat. Energy 2, 17135 (2017).

[10] Y.-W. Jang, S. Lee, K. M. Yeom, K. Jeong, K. Choi, M. Choi, J. H. Noh, Intact 2D/3D halide junction perovskite solar cells via solid-phase in-plane growth. Nat. Energy 6, 63–71 (2021).

[11] F. Zhang, H. Lu, J. Tong, J. J. Berry, M. C. Beard, K. Zhu, Advances in two-dimensional organic-inorganic hybrid perovskites. Energ. Environ. Sci. 13, 1154–1186 (2020).

[12] J. J. Yoo, G. Seo, M. R. Chua, T. G. Park, Y. Lu, F. Rotermund, Y.-K. Kim, C. S. Moon, N. J. Jeon, J.-P. Correa-Baena, V. Bulović, S. S. Shin, M. G. Bawendi, J. Seo, Efficient perovskite solar cells via improved carrier management. Nature 590, 587–593 (2021). 33627807 10.1038/s41586-021-03285-w

[13] F. Zhang, S. Y. Park, C. Yao, H. Lu, S. P. Dunfield, C. Xiao, S. Ulǐná, X. Zhao, L. D. Hill, X. Chen, X. Wang, L. E. Mundt, K. H. Stone, L. T. Schelhas, G. Teeter, S. Parkin, E. L. Ratcliff, Y.-L. Loo, J. J. Berry, M. C. Beard, Y. Yan, B. W. Larson, K. Zhu, Metastable Dion-Jacobson 2D structure enables efficient and stable perovskite solar cells. Science 375, 71–76 (2022). 34822309 10.1126/science.abj2637

[14] S. Sidhik, Y. Wang, M. De Siena, R. Asadpour, A. J. Torma, T. Terlier, K. Ho, W. Li, A. B. Puthirath, X. Shuai, A. Agrawal, B. Traore, M. Jones, R. Giridharagopal, P. M. Ajayan, J. Strzalka, D. S. Ginger, C. Katan, M. A. Alam, J. Even, M. G. Kanatzidis, A. D. Mohite, Deterministic fabrication of 3D/2D perovskite bilayer stacks for durable and efficient solar cells. Science 377, 1425–1430 (2022). 36137050 10.1126/science.abq7652

[15] H. Chen, S. Teale, B. Chen, Y. Hou, L. Grater, T. Zhu, K. Bertens, S. M. Park, H. R. Atapattu, Y. Gao, M. Wei, A. K. Johnston, Q. Zhou, K. Xu, D. Yu, C. Han, T. Cui, E. H. Jung, C. Zhou, W. Zhou, A. H. Proppe, S. Hoogland, F. Laquai, T. Filleter, K. R. Graham, Z. Ning, E. H. Sargent, Quantum-size-tuned heterostructures enable efficient and stable inverted perovskite solar cells. Nat. Photonics 16, 352–358 (2022).

[16] R. Azmi, E. Ugur, A. Seitkhan, F. Aljamaan, A. S. Subbiah, J. Liu, G. T. Harrison, M. I. Nugraha, M. K. Eswaran, M. Babics, Y. Chen, F. Xu, T. G. Allen, A. U. Rehman, C.-L. Wang, T. D. Anthopoulos, U. Schwingenschlögl, M. De Bastiani, E. Aydin, S. De Wolf, Damp heat–stable perovskite solar cells with tailored-dimensionality 2D/3D heterojunctions. Science 376, 73–77 (2022). 35175829 10.1126/science.abm5784

[17] J. Xue, R. Wang, X. Chen, C. Yao, X. Jin, K.-L. Wang, W. Huang, T. Huang, Y. Zhao, Y. Zhai, D. Meng, S. Tan, R. Liu, Z.-K. Wang, C. Zhu, K. Zhu, M. C. Beard, Y. Yan, Y. Yang, Reconfiguring the band-edge states of photovoltaic perovskites by conjugated organic cations. Science 371, 636–640 (2021). 33542138 10.1126/science.abd4860

[18] Y. Gao, E. Shi, S. Deng, S. B. Shiring, J. M. Snaider, C. Liang, B. Yuan, R. Song, S. M. Janke, A. Liebman-Peláez, P. Yoo, M. Zeller, B. W. Boudouris, P. Liao, C. Zhu, V. Blum, Y. Yu, B. M. Savoie, L. Huang, L. Dou, Molecular engineering of organic–inorganic hybrid perovskites quantum wells. Nat. Chem. 11, 1151–1157 (2019). 31712613 10.1038/s41557-019-0354-2

[19] H. Tsai, R. Asadpour, J.-C. Blancon, C. C. Stoumpos, J. Even, P. M. Ajayan, M. G. Kanatzidis, M. A. Alam, A. D. Mohite, W. Nie, Design principles for electronic charge transport in solution-processed vertically stacked 2D perovskite quantum wells. Nat. Commun. 9, 2130 (2018). 29849026 10.1038/s41467-018-04430-2 PMC5976721

[20] T. He, S. Li, Y. Jiang, C. Qin, M. Cui, L. Qiao, H. Xu, J. Yang, R. Long, H. Wang, M. Yuan, Reduced-dimensional perovskite photovoltaics with homogeneous energy landscape. Nat. Commun. 11, 1672 (2020). 32246083 10.1038/s41467-020-15451-1 PMC7125147

[21] K. Ma, H. R. Atapattu, Q. Zhao, Y. Gao, B. P. Finkenauer, K. Wang, K. Chen, S. M. Park, A. H. Coffey, C. Zhu, L. Huang, K. R. Graham, J. Mei, L. Dou, Multifunctional conjugated ligand engineering for stable and efficient perovskite solar cells. Adv. Mater. 33, 2100791 (2021). 10.1002/adma.202100791 34219297

[22] A. Bala, V. Kumar, Effects of Cl and F substitution in phenylethylammonium spacer cations on stability, structure, and optical properties of 2D–3D ruddlesden–Popper perovskite layers. ACS Appl. Energy Mater. 4, 1860–1867 (2021).

[23] J. V. Passarelli, D. J. Fairfield, N. A. Sather, M. P. Hendricks, H. Sai, C. L. Stern, S. I. Stupp, Enhanced out-of-plane conductivity and photovoltaic performance in n = 1 layered perovskites through organic cation design. J. Am. Chem. Soc. 140, 7313–7323 (2018). 29869499 10.1021/jacs.8b03659

[24] G. Liu, X.-X. Xu, S. Xu, L. Zhang, H. Xu, L. Zhu, X. Zhang, H. Zheng, X. Pan, Passivation effect of halogenated benzylammonium as a second spacer cation for improved photovoltaic performance of quasi-2D perovskite solar cells. J. Mater. Chem. A 8, 5900–5906 (2020).

[25] J. Hu, I. W. H. Oswald, S. J. Stuard, M. M. Nahid, N. Zhou, O. F. Williams, Z. Guo, L. Yan, H. Hu, Z. Chen, X. Xiao, Y. Lin, Z. Yang, J. Huang, A. M. Moran, H. Ade, J. R. Neilson, W. You, Synthetic control over orientational degeneracy of spacer cations enhances solar cell efficiency in two-dimensional perovskites. Nat. Commun. 10, 1276 (2019). 30894519 10.1038/s41467-019-08980-x PMC6427015

[26] D. Kim, H. J. Jung, I. J. Park, B. W. Larson, S. P. Dunfield, C. Xiao, J. Kim, J. Tong, P. Boonmongkolras, S. G. Ji, F. Zhang, S. R. Pae, M. Kim, S. B. Kang, V. Dravid, J. J. Berry, J. Y. Kim, K. Zhu, D. H. Kim, B. Shin, Efficient, stable silicon tandem cells enabled by anion-engineered wide-bandgap perovskites. Science 368, 155–160 (2020). 32217753 10.1126/science.aba3433

[27] Y. Gao, Z. Wei, P. Yoo, E. Shi, M. Zeller, C. Zhu, P. Liao, L. Dou, Highly stable lead-free perovskite field-effect transistors incorporating linear π-conjugated organic ligands. J. Am. Chem. Soc. 141, 15577–15585 (2019). 31525969 10.1021/jacs.9b06276

[28] A. H. Proppe, R. Quintero-Bermudez, H. Tan, O. Voznyy, S. O. Kelley, E. H. Sargent, Synthetic control over quantum well width distribution and carrier migration in low-dimensional perovskite photovoltaics. J. Am. Chem. Soc. 140, 2890–2896 (2018). 29397693 10.1021/jacs.7b12551

[29] M. Daboczi, S. R. Ratnasingham, L. Mohan, C. Pu, I. Hamilton, Y.-C. Chin, M. A. Mclachlan, J.-S. Kim, Optimal interfacial band bending achieved by fine energy level tuning in mixed-halide perovskite solar cells. ACS Energy Lett. 6, 3970–3981 (2021).

[30] Y. Zhao, T. Heumueller, J. Zhang, J. Luo, O. Kasian, S. Langner, C. Kupfer, B. Liu, Y. Zhong, J. Elia, A. Osvet, J. Wu, C. Liu, Z. Wan, C. Jia, N. Li, J. Hauch, C. J. Brabec, A bilayer conducting polymer structure for planar perovskite solar cells with over 1,400 hours operational stability at elevated temperatures. Nat. Energy 7, 144–152 (2022).

[31] Y. Wang, L. Duan, Z. Hameiri, M. Zhang, X. Liu, Y. Bai, X. Hao, PTAA as efficient hole transport materials in perovskite solar cells: A review. Sol. RRL 6, 2200234 (2022).

[32] F. M. Rombach, S. A. Haque, T. J. Macdonald, Lessons learned from spiro-OMeTAD and PTAA in perovskite solar cells. Energ. Environ. Sci. 14, 5161–5190 (2021).

[33] W. S. Yang, B. W. Park, E. H. Jung, N. J. Jeon, Y. C. Kim, D. U. Lee, S. S. Shin, J. Seo, E. K. Kim, J. H. Noh, S. I. Seok, Iodide management in formamidinium-lead-halide-based perovskite layers for efficient solar cells. Science 356, 1376–1379 (2017). 28663498 10.1126/science.aan2301

[34] M. Jeong, I. W. Choi, E. M. Go, Y. Cho, M. Kim, B. Lee, S. Jeong, Y. Jo, H. W. Choi, J. Lee, J.-H. Bae, S. K. Kwak, D. S. Kim, C. Yang, Stable perovskite solar cells with efficiency exceeding 24.8% and 0.3-V voltage loss. Science 369, 1615–1620 (2020). 32973026 10.1126/science.abb7167

[35] H. Ren, S. Yu, L. Chao, Y. Xia, Y. Sun, S. Zuo, F. Li, T. Niu, Y. Yang, H. Ju, B. Li, H. Du, X. Gao, J. Zhang, J. Wang, L. Zhang, Y. Chen, W. Huang, Efficient and stable Ruddlesden–Popper perovskite solar cell with tailored interlayer molecular interaction. Nat. Photonics 14, 154–163 (2020).

[36] Z. Liu, L. Qiu, L. K. Ono, S. He, Z. Hu, M. Jiang, G. Tong, Z. Wu, Y. Jiang, D.-Y. Son, Y. Dang, S. Kazaoui, Y. Qi, A holistic approach to interface stabilization for efficient perovskite solar modules with over 2,000-hour operational stability. Nat. Energy 5, 596–604 (2020).

[37] M. Stolterfoht, C. M. Wolff, J. A. Márquez, S. Zhang, C. J. Hages, D. Rothhardt, S. Albrecht, P. L. Burn, P. Meredith, T. Unold, D. Neher, Visualization and suppression of interfacial recombination for high-efficiency large-area pin perovskite solar cells. Nat. Energy 3, 847–854 (2018).

[38] J. Wang, W. Fu, S. Jariwala, I. Sinha, A. K.-Y. Jen, D. S. Ginger, Reducing surface recombination velocities at the electrical contacts will improve perovskite photovoltaics. ACS Energy Lett. 4, 222–227 (2019).

[39] Z. Ni, H. Jiao, C. Fei, H. Gu, S. Xu, Z. Yu, G. Yang, Y. Deng, Q. Jiang, Y. Liu, Y. Yan, J. Huang, Evolution of defects during the degradation of metal halide perovskite solar cells under reverse bias and illumination. Nat. Energy 7, 65–73 (2022).

[40] L. J. Farrugia, WinGX and ORTEP for Windows: An update. J. Appl. Cryst. 45, 849–854 (2012).

[41] C. B. Hübschle, G. M. Sheldrick, B. Dittrich, ShelXle: A Qt graphical user interface for SHELXL. J. Appl. Cryst. 44, 1281–1284 (2011). 22477785 10.1107/S0021889811043202 PMC3246833

[42] G. M. Sheldrick, Crystal structure refinement with SHELXL. Acta Crystallogr. C 71, 3–8 (2015). 10.1107/S2053229614024218 PMC4294323 25567568

[43] G. M. Sheldrick, SHELXT–integrated space-group and crystal-structure determination. Acta Crystallogr. Sect. A 71, 3–8 (2015). 10.1107/S2053273314026370 PMC4283466 25537383

