# Supplementary material for: Holistic energy landscape management in 2D/3D heterojunction via molecular engineering for efficient perovskite solar cells
Source: Sci Adv. 2023 Jun 7;9(23):eadg0032. doi: 10.1126/sciadv.adg0032 (PMC10246895; doi:10.1126/sciadv.adg0032)
Supplement: Supplementary file 1 — Supplementary Text Figs. S1 to S32 Tables S1 to S7 References [file sciadv.adg0032_sm.pdf]

Science Advances
AAAS

Supplementary Materials for

Holistic energy landscape management in 2D/3D heterojunction via molecular engineering for efficient perovskite solar cells

Ke Ma *et al.*

Corresponding author: Letian Dou, dou10@purdue.edu

Sci. Adv. **9**, eadg0032 (2023)
DOI: 10.1126/sciadv.adg0032

This PDF file includes:

Supplementary Text
Figs. S1 to S32
Tables S1 to S7
References

1. Conjugated organic ligand synthesis

F4TmI Synthesis

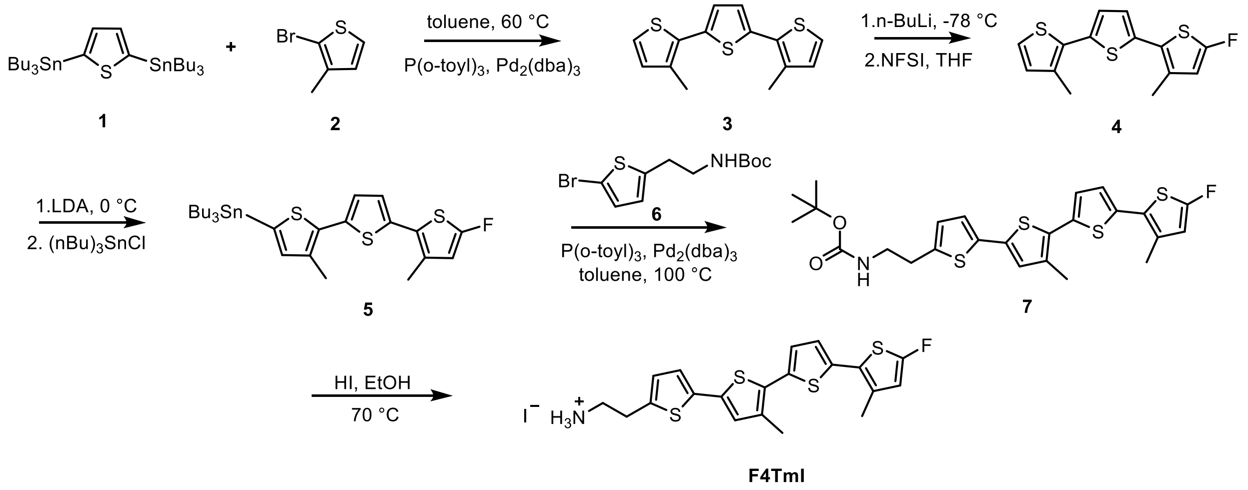

3,3''-Dimethyl-2,2':5',2''-terthiophene (**3**):

2,5-bis(tributylstannyl)thiophene (**1**, 2.6 g, 7.8 mmol), 2-bromo-3-methylthiophene (**2**, 1.4 g, 3.9 mmol), tris(dibenzylideneacetone)dipalladium(0) (72 mg, 0.078 mmol, 2%) and tri(*o*-tolyl)phosphine (95 mg, 0.31 mmol, 8%) was mixed and then degassed three time via Schlenk tube. 60 ml dry toluene was added via syringe and the mixture was stirred at 60 °C for 4 hours. After the reaction was cooled to room temperature, water was added. Diethyl ether was used to extract the product in a separatory funnel three times and the organic phase was further washed with brine. All the organic layers were combined and dried with magnesium sulfate. After filtration, the solvent was removed by rotary evaporation and the crude product was further purified by silica column with pure hexane. In the end, 0.79 g of light-yellow solid was obtained in a yield of 71%. ^{1}H NMR (400 MHz, $CDCl_3$) δ 7.15 (d, J = 5.1 Hz, 2H), 7.08 (s, 2H), 6.89 (d, J = 5.0 Hz, 2H), 2.42 (s, 6H).

5-Fluoro-3,3''-dimethyl-2,2':5',2''-terthiophene (**4**):

To a solution of **3** (7.2 mmol, 2 g) dissolved in 112 ml dry THF, 2.5 M n-butyl lithium (7.4 mmol, 3 ml) was added dropwise via syringe at -78 °C in dry ice/acetone bath in argon environment. Then the dry ice was removed, and the mixture was stirred for 1.5 hour during warm up. The mixture was then cooled to -78 °C again and N-fluorobenzenesulfonimide (NFSI, 7.9 mmol, 2.5g) dissolved in 10 ml dry THF was added to the reaction mixture. The reaction was then stirred at

room temperature overnight and water was added to quench the reaction. Dichloromethane was used to extract the product in a separatory funnel three times and the organic phase was further washed with brine. All the organic layers were combined and dried with magnesium sulfate. The solvent was removed by rotary evaporation and the crude product was further purified by column chromatography with pure hexane. 1.1 g yellow solid was obtained in a yield of 53%. ^{1}H NMR (400 MHz, $CDCl_3$) δ 7.15 (d, *J* = 5.1 Hz, 1H), 7.05 (d, *J* = 3.8 Hz, 1H), 6.98 (d, *J* = 3.8 Hz, 1H), 6.91 – 6.84 (m, 1H), 6.30 (d, *J* = 2.3 Hz, 1H), 2.41 (s, 3H), 2.31 (s, 3H).

Tributyl(5''-fluoro-3,3''-dimethyl-[2,2':5',2''-terthiophen]-5-yl)stannane (**5**)

To a solution of **4** (1.5 mmol, 447 mg) dissolved in 16 ml dry THF, 2.0 M lithium diisopropylamide in hexane (1.67 mmol, 0.84 ml) was added dropwise via syringe at 0 °C in ice bath. Then the ice bath was removed, and the mixture was stirred for 1.5 hour during warm up. The mixture was then cooled to 0 °C again and tributyltin chloride (1.67 mmol, 0.45 ml) was added to the reaction mixture. The reaction was then stirred at room temperature overnight and water was added to quench the reaction. Dichloromethane was used to extract the product in a separatory funnel three times and the organic phase was further washed with brine. All the organic layers were combined and dried with magnesium sulfate. The solvent was removed by rotary evaporation and the crude product was used without further purification assuming 67% conversion.

tert-butyl (2-(5'''-fluoro-3''',4'-dimethyl-[2,2':5',2'':5'',2'''-quaterthiophen]-5-yl)ethyl)carbamate (**7**)

Tributyl(5''-fluoro-3,3''-dimethyl-[2,2':5',2''-terthiophen]-5-yl)stannane (**5**, 0.89 g, 1.52 mmol), tert-butyl (2-(5-bromothiophen-2-yl)ethyl)carbamate (**6**, 0.31 g, 1.0 mmol), tris(dibenzylideneacetone)-dipalladium(0) (27.8 mg, 0.030 mmol, 2%) and tri(*o*-tolyl)phosphine (37 mg, 0.12 mmol, 8%) was mixed and then degassed three time via Schlenk tube. 15.3 ml dry toluene was added via syringe and the mixture was stirred overnight at 100 °C. After the reaction was cooled to room temperature, water was added. Dichloromethane was used to extract the product in a separatory funnel three times and the organic phase was further washed with brine. All the organic layers were combined and dried with magnesium sulfate. After filtration, the solvent was removed by rotary evaporation and the crude product was further purified by silica column with hexane and ethyl acetate in 5:1 ratio. In the end, 0.35 g of light-yellow solid was obtained in a yield of 65%. ^{1}H NMR (400 MHz, CDCl3) δ 7.05 (d, J = 3.8 Hz, 1H), 6.98 (dd, J =

3.7, 2.4 Hz, 2H), 6.90 (s, 1H), 6.73 (dt, J = 3.6, 0.9 Hz, 1H), 6.30 (d, J = 2.3 Hz, 1H), 4.68 (s, 1H), 3.41 (d, J = 6.8 Hz, 2H), 2.99 (s, 1H), 2.35 (d, J = 27.6 Hz, 5H), 1.45 (s, 9H).

2-(5'''-fluoro-3''',4'-dimethyl-[2,2':5',2'':5'',2'''-quaterthiophen]-5-yl)ethan-1-aminium iodide (**F4TmI**)

To a solution of **7** (180 mg, 0.35 mmol) in 10 ml ethanol was added hydroiodic acid (57 wt%, 0.09 ml, 0.7 mmol) and the reaction mixture was stirred at 70°C for 3 hours with argon flow. After the reaction was completed, the solvent was partially removed on a Rotovap until yellow solids precipitate out. Diethyl ether was added to the mixture to further precipitate out the yellow solid. The mixture was sonicated for 5 minutes, filtered, and washed with diethyl ether. Finally, 157 mg of pure F4Tm was obtained in a yield of 83%. ^{1}H NMR (400 MHz, DMSO) δ 7.79 (s, 3H), 7.44 – 7.06 (m, 4H), 6.96 (d, J = 3.6 Hz, 1H), 6.71 (d, J = 2.6 Hz, 1H), 3.08 (s, 4H), 2.37 (s, 3H), 2.30 (s, 3H). ^{13}C NMR (101 MHz, DMSO) δ 139.21, 135.51, 135.40, 135.00, 134.59, 134.34, 131.75, 128.51, 127.85, 127.21, 126.48, 124.63, 118.86, 113.03, 112.94, 27.79, 16.00, 15.77. HR-MS (ESI) Expected 420.0379 [M - I]+ Observed 420.0363

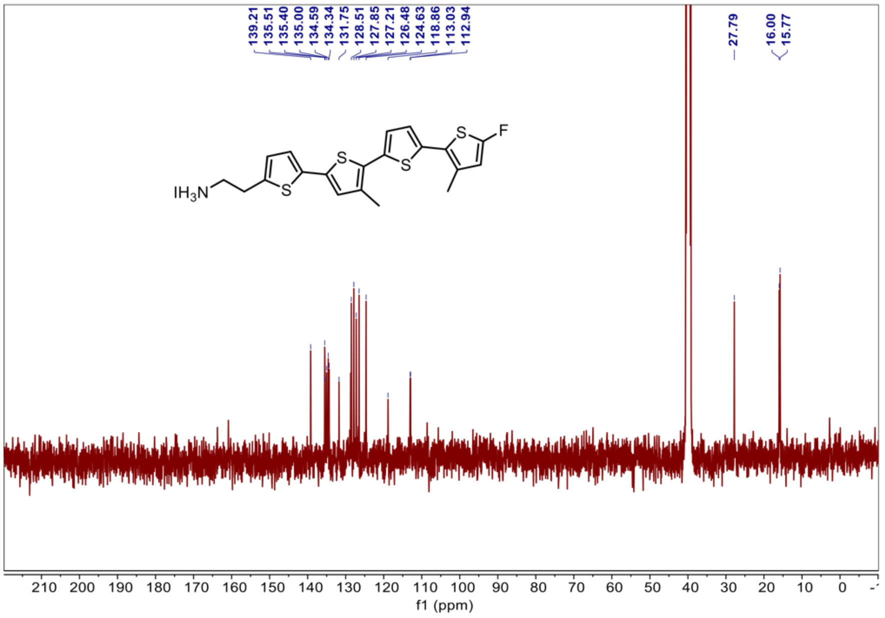

^{13}C NMR (101 MHz, DMSO-d6) of F4TmI

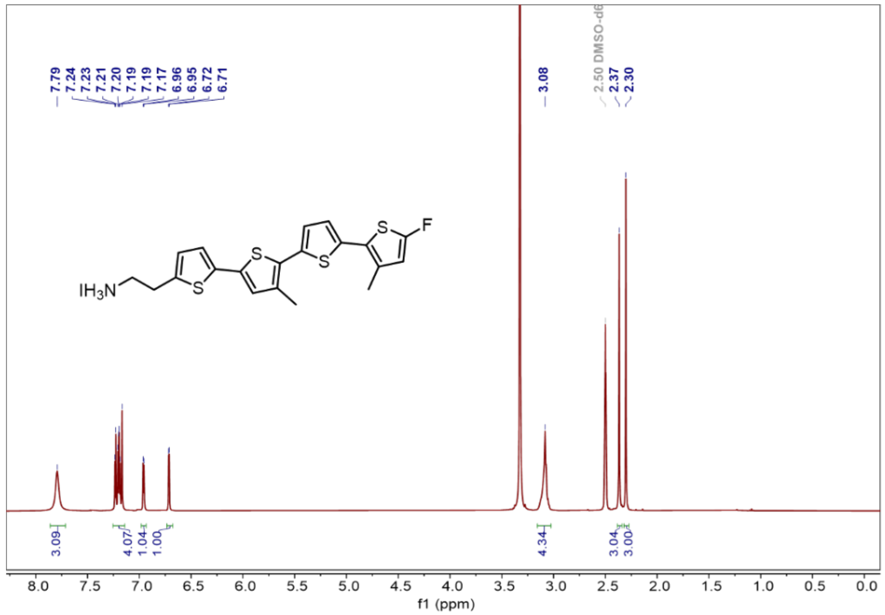

^{1}H NMR (400 MHz, DMSO-d6) of F4TmI

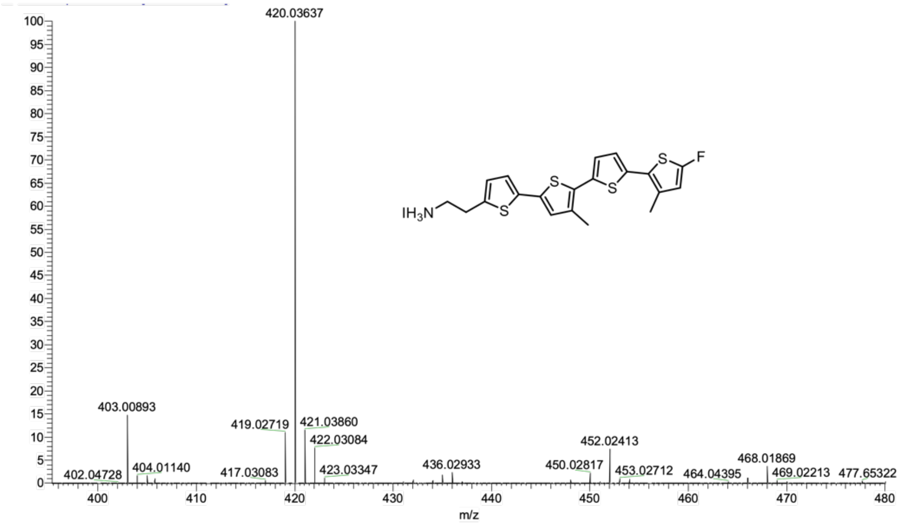

HR-MS (ESI) of F4TmI

Cl4TmI Synthesis

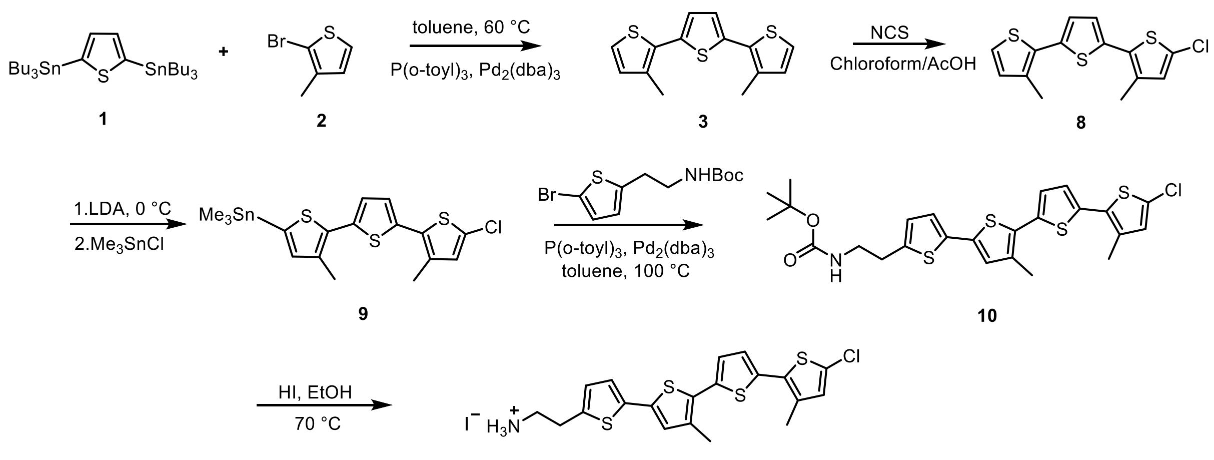

Cl4TmI

5-chloro-3,3''-dimethyl-2,2':5',2''-terthiophene (**8**):

To a solution of **3** (3.62 mmol, 1g) dissolved in 50 ml chloroform and 50 ml acetic acid, *N*-chlorosuccinimide (NCS, 3.8 mmol, 0.5g) was added to the reaction mixture. The reaction was then stirred at room temperature for 71 hours. Dichloromethane was used to extract the product in a separatory funnel three times and the organic phase was further washed with brine. All the organic layers were combined and dried with magnesium sulfate. The solvent was removed by rotary evaporation and the crude product was further purified by column chromatography with pure hexane. 0.57 g yellow solid was obtained in a yield of 50%. ^{1}H NMR (400 MHz, $CDCl_3$) δ 7.15 (d, J = 5.1 Hz, 1H), 7.06 (d, J = 3.8 Hz, 1H), 7.01 (d, J = 3.8 Hz, 1H), 6.91 – 6.84 (m, 1H), 6.74 – 6.69 (m, 1H), 2.41 (s, 3H), 2.34 (s, 3H).

(5''-chloro-3,3''-dimethyl-[2,2':5',2''-terthiophen]-5-yl)trimethylstannane (**9**)

To a solution of **8** (1.64 mmol, 510 mg) dissolved in 17 ml dry THF, 2.0 M lithium diisopropylamide in hexane (1.80 mmol, 0.9 ml) was added dropwise via syringe at 0 °C in ice bath. Then the ice bath was removed, and the mixture was stirred for 1.5 hour during warm up. The mixture was then cooled to 0 °C again and trimethyltin chloride (1 M, 1.80 mmol, 1.8 ml) was added to the reaction mixture. The reaction was then stirred at room temperature overnight and water was added to quench the reaction. Dichloromethane was used to extract the product in a separatory funnel three times and the organic phase was further washed with brine. All the organic layers were combined and dried with magnesium sulfate. The solvent was removed by rotary evaporation and the crude product was used without further purification assuming 70% conversion.

tert-butyl (2-(5'''-chloro-3''',4'-dimethyl-[2,2':5',2'':5'',2'''-quaterthiophen]-5-yl)ethyl)carbamate (**10**)

(5''-chloro-3,3''-dimethyl-[2,2':5',2''-terthiophen]-5-yl)trimethylstannane (**9**, 0.78 g, 1.64 mmol), tert-butyl (2-(5-bromothiophen-2-yl)ethyl)carbamate (**6**, 0.35 g, 1.15 mmol), tris(dibenzylideneacetone)-dipalladium(0) (30 mg, 0.033 mmol, 2%) and tri(*o*-tolyl)phosphine (40 mg, 0.13 mmol, 8%) was mixed and then degassed three time via Schlenk tube. 16 ml dry toluene was added via syringe and the mixture was stirred overnight at 100 °C. After the reaction was cooled to room temperature, water was added. Dichloromethane was used to extract the product in a separatory funnel three times and the organic phase was further washed with brine. All the organic layers were combined and dried with magnesium sulfate. After filtration, the solvent was removed by rotary evaporation and the crude product was further purified by silica column with hexane and ethyl acetate in 5:1 ratio. In the end, 0.28 g of light-yellow solid was obtained in a yield of 46%. ^{1}H NMR (400 MHz, $CDCl_3$) δ 7.07 (d, J = 3.8 Hz, 1H), 7.00 (dd, J = 10.5, 3.7 Hz, 2H), 6.91 (s, 1H), 6.75 – 6.70 (m, 2H), 4.68 (s, 1H), 3.41 (d, J = 6.8 Hz, 2H), 2.99 (t, J = 6.7 Hz, 2H), 2.37 (d, J = 17.8 Hz, 6H), 1.45 (s, 9H).

2-(5'''-chloro-3''',4'-dimethyl-[2,2':5',2'':5'',2'''-quaterthiophen]-5-yl)ethan-1-aminium iodide (**Cl4TmI**)

To a solution of **10** (167 mg, 0.31 mmol) in 7 ml ethanol was added hydroiodic acid (57 wt%, 0.08 ml, 0.6 mmol) and the reaction mixture was stirred at 77°C for 3 hours with argon flow. After the reaction was completed, the solvent was partially removed on a Rotovap until yellow solids precipitate out. Diethyl ether was added to the mixture to further precipitate out the yellow solid. The mixture was sonicated for 5 minutes, filtered, and washed with diethyl ether. Finally, 162 mg of pure Cl4TmI was obtained in a yield of 92%. ^{1}H NMR (400 MHz, DMSO) δ 7.74 (s, 3H), 7.27 – 7.20 (m, 3H), 7.17 (s, 1H), 7.08 (s, 1H), 6.96 (d, J = 3.7 Hz, 1H), 3.16 – 2.94 (m, 4H), 2.37 (s, 3H), 2.33 (s, 3H). ^{13}C NMR (101 MHz, DMSO) δ 139.24, 135.78, 135.64, 134.98, 134.51, 134.45, 134.14, 131.73, 129.07, 128.61, 128.53, 127.86, 127.34, 126.56, 126.39, 124.66, 27.79, 15.78, 15.64. HR-MS (ESI) Expected 436.0089 [M - I]+ Observed 436.0077

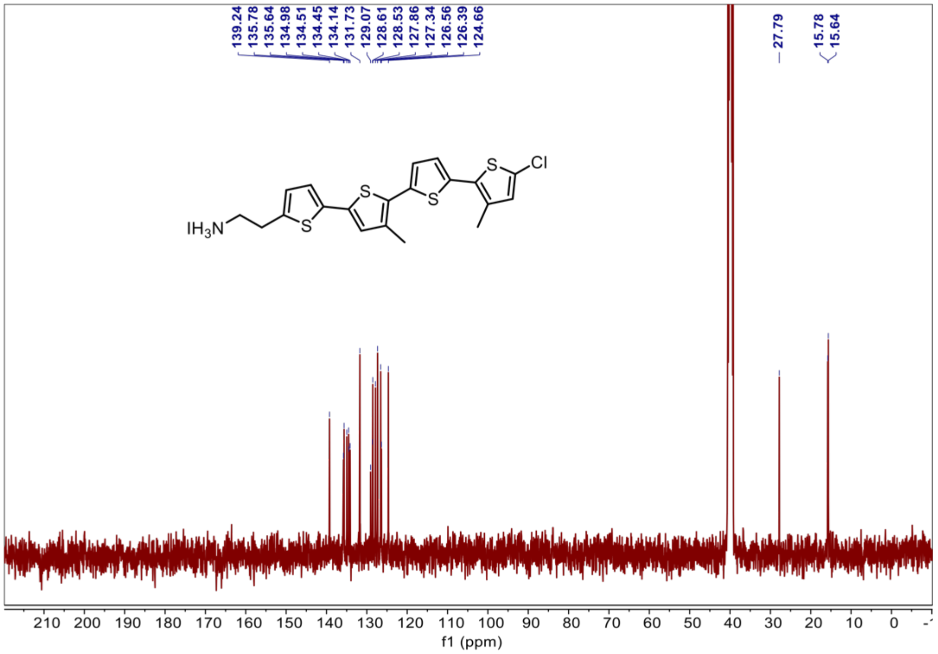

^{13}C NMR (101 MHz, DMSO-d6) of Cl4TmI

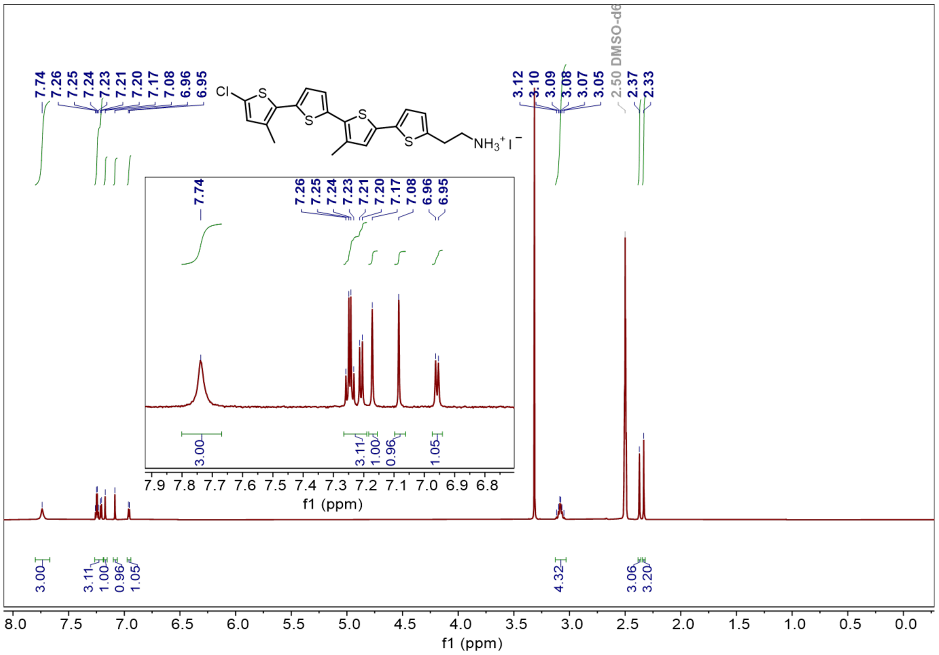

^{1}H NMR (400 MHz, DMSO-d6) of Cl4TmI

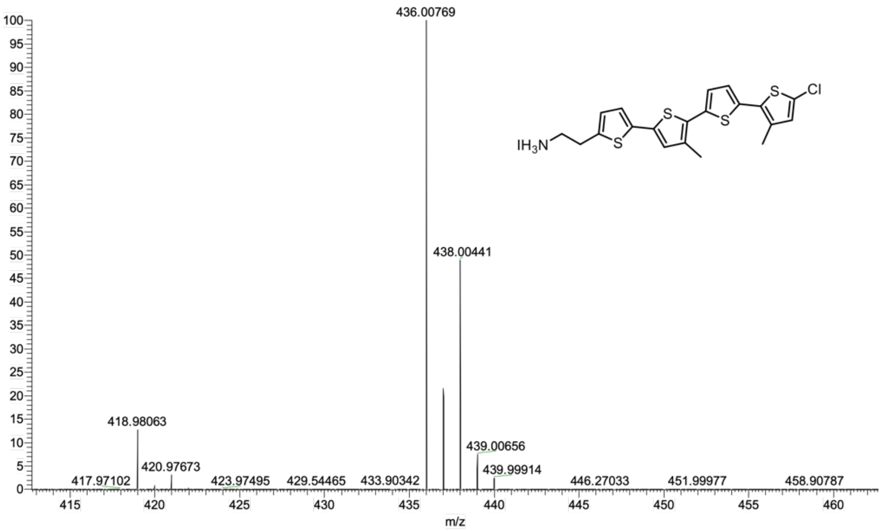

HR-MS (ESI) of Cl4TmI

Br4TmI Synthesis

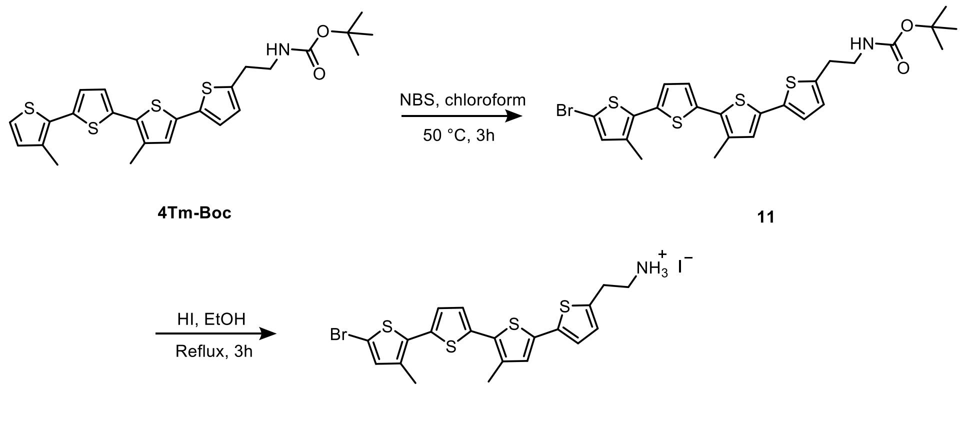

The synthesis of Br4Tm was based on 4Tm-Boc and details of 4Tm-Boc synthesis can be found in our previous reports.

tert-butyl (2-(5'''-bromo-3''',4'-dimethyl-[2,2':5',2'':5'',2'''-quaterthiophen]-5-yl)ethyl)carbamate (**11**)

To a solution of **4Tm-Boc** (0.50 mmol, 0.25 g) dissolved in 4 ml chloroform, *N*-bromosuccinimide (NBS, 0.51 mmol, 92 mg) was added to the reaction mixture. The reaction was then stirred at room temperature for 22 hours and then stirred at 50°C for 2 hours. Dichloromethane was used to extract the product in a separatory funnel three times and the organic phase was further washed with brine. All the organic layers were combined and dried with magnesium sulfate. The solvent was removed by rotary evaporation and the crude product was further purified by column chromatography with

hexane and ethyl acetate in 5:1 ratio. 0.19 g yellow solid was obtained in a yield of 65%. ^{1}H NMR (400 MHz, $CDCl_3$) δ 7.06 (d, J = 3.8 Hz, 1H), 7.02 (d, J = 3.8 Hz, 1H), 6.99 (d, J = 3.6 Hz, 1H), 6.90 (s, 1H), 6.86 (s, 1H), 6.73 (dt, J = 3.6, 0.9 Hz, 1H), 4.68 (s, 1H), 3.41 (d, J = 6.9 Hz, 2H), 2.99 (t, J = 6.6 Hz, 2H), 2.37 (d, J = 9.9 Hz, 6H), 1.45 (s, 9H).

2-(5'''-bromo-3''',4'-dimethyl-[2,2':5',2'':5'',2'''-quaterthiophen]-5-yl)ethan-1-aminium (**Br4TmI**)

To a solution of **11** (66 mg, 0.12 mmol) in 10 ml ethanol was added hydroiodic acid (57 wt%, 33 ul, 0.25 mmol) and the reaction mixture was stirred at 80 °C for 4 hours with argon flow. After the reaction was completed, the solvent was partially removed on a Rotovap until yellow solids precipitate out. Diethyl ether was added to the mixture to further precipitate out the yellow solid. The mixture was sonicated for 5 minutes, filtered, and washed with diethyl ether. Finally, 66 mg of pure Br4TmI was obtained in a yield of 94%. ^{1}H NMR (400 MHz, DMSO) δ 7.79 (s, 3H), 7.28 – 7.19 (m, 3H), 7.17 (d, J = 1.3 Hz, 2H), 6.96 (dd, J = 3.7, 1.3 Hz, 1H), 3.18 – 2.83 (m, 4H), 2.36 (dd, J = 9.5, 1.3 Hz, 6H). ^{13}C NMR (101 MHz, DMSO) δ 139.24, 135.79, 135.63, 135.45, 135.22, 134.98, 134.44, 134.18, 131.72, 128.63, 128.53, 127.86, 127.29, 126.56, 124.66, 109.82, 27.79, 15.79, 15.46. HR-MS (ESI) Expected 479.9578 [M - I]+ Observed 479.9569

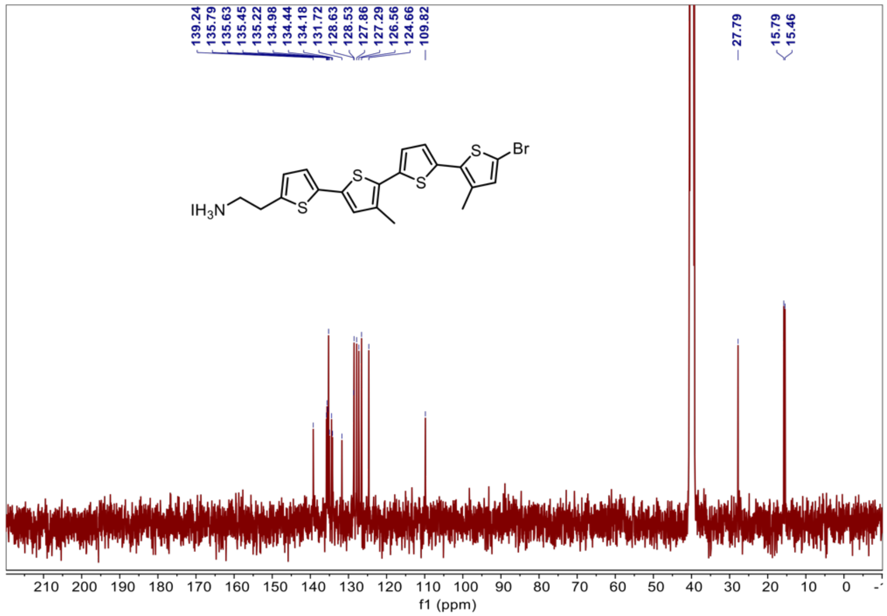

^{13}C NMR (101 MHz, DMSO-d6) of Br4TmI

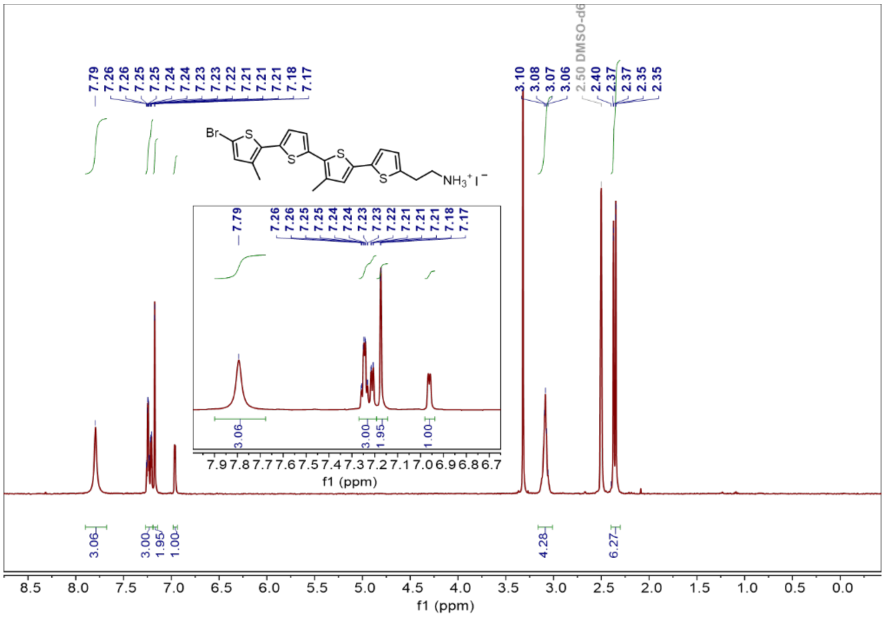

^{1}H NMR (400 MHz, DMSO-d6) of Br4TmI

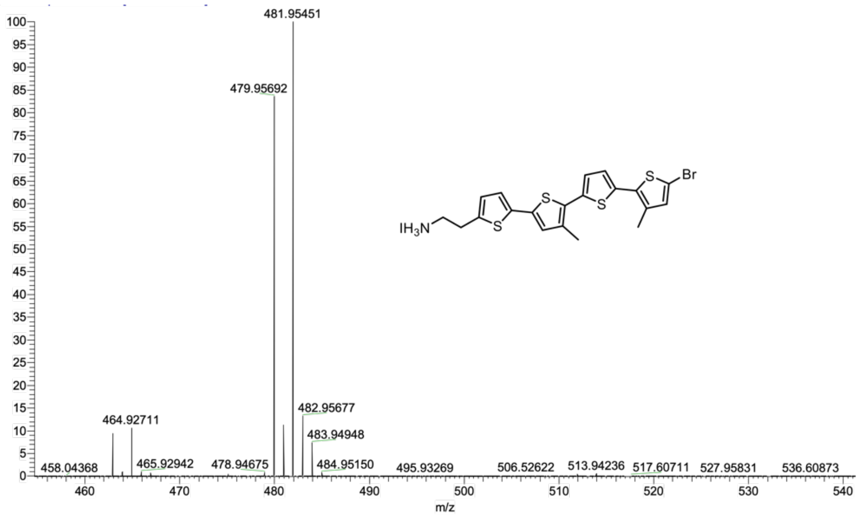

HR-MS (ESI) of Br4TmI

2. Supplementary figures

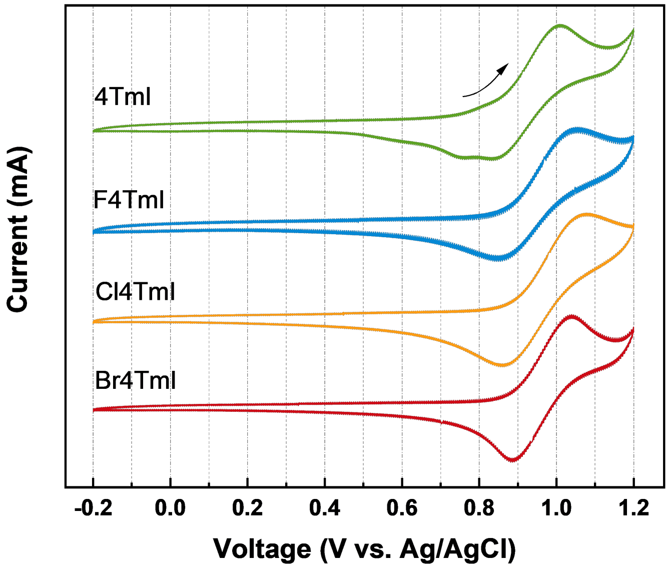

Fig. S1. Cyclic voltammetry measurement of different ligands, including 4TmI, F4TmI, Cl4TmI and Br4TmI. To avoid the redox peaks from iodide counter ions overlapping with the redox peaks from conjugated ligands, a tert-butyl carbamate (Boc) terminal group was used to replace the iodide counter ion without affecting the oxidation potentials of ligands. For 4TmI, one extra reduction peak is observed due to the instability of oxidized 4TmI, while F4TmI, Cl4TmI and Br4TmI show excellent redox reversibility with symmetric redox peaks.

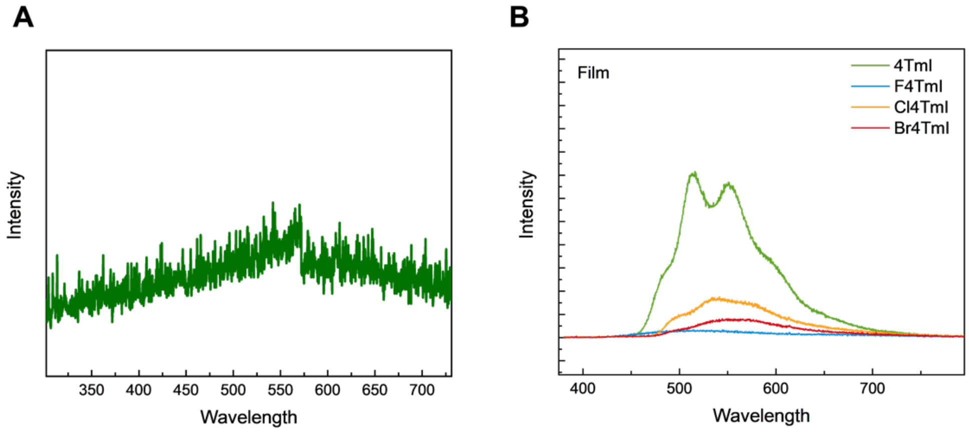

Fig. S2. **Steady state photoluminescence spectra of thin films.** (**A**) $(F4Tm)_2PbI_4$ 2D perovskite thin film (apply to all others halogen-4Tm). (**B**) Thin films formed with pure organic ligands.

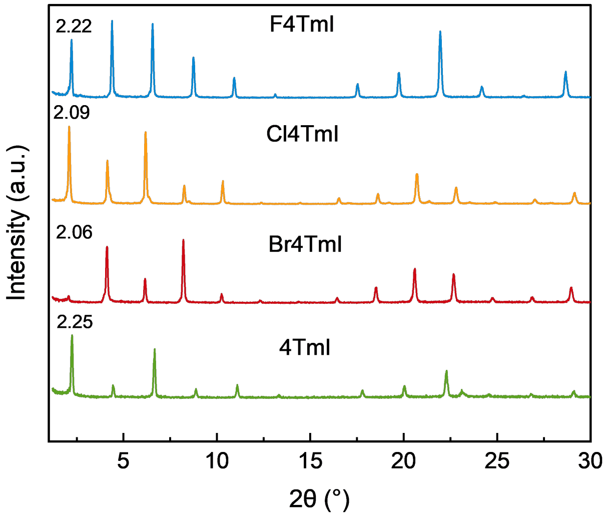

Fig. S3. XRD patterns of thin films formed with pure ligand aggregation. All the ligand thin films were annealed at 150 °C.

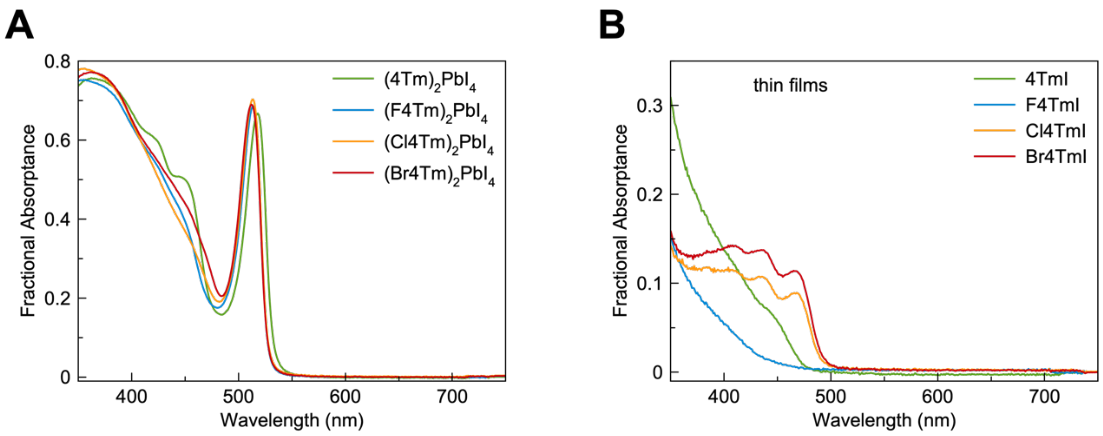

Fig. S4. Absorption spectra of thin films. (**A**) 2D perovskite thin films formed with different ligands. (**B**) Pure ligand thin films.

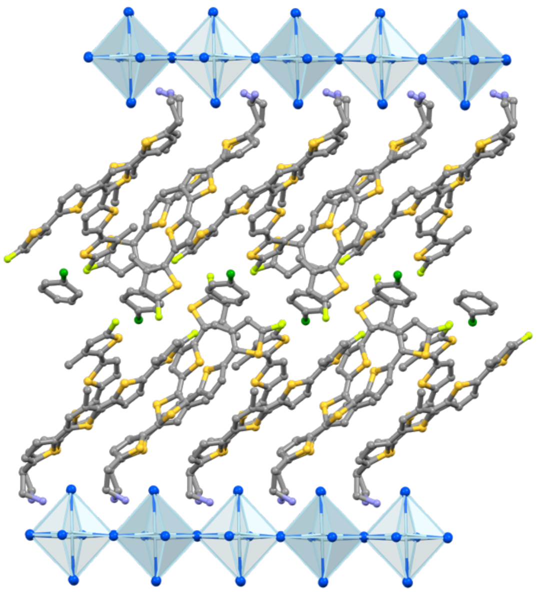

Fig. S5. Single crystal structure of $(F4Tm)_2PbI_4$ with chlorobenzene intercalation.

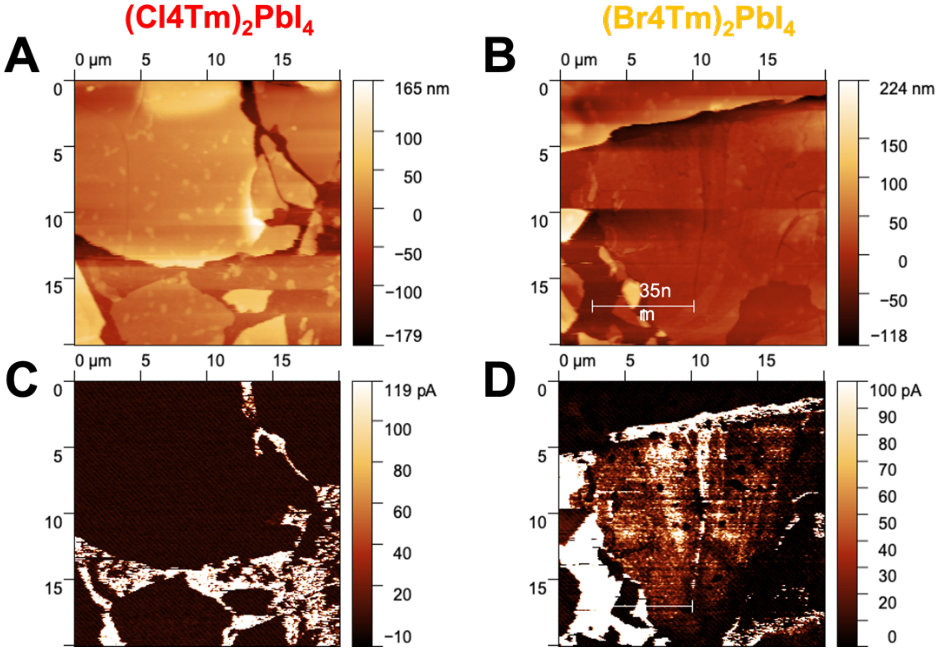

Fig. S6. The AFM images and corresponding conductive-AFM images of 2D perovskite single crystals. The AFM images (**A** and **B**) and corresponding conductive-AFM (cAFM) maps (**C** and **D**) of 2D single crystals $(Cl4Tm)_2PbI_4$ (**A** and **C**) and $(Br4Tm)_2PbI_4$ (**B** and **D**). The labels in the AFM images indicate the thickness of 2D crystals. The single crystal samples were prepared by peeling off a thin layer of 2D crystal from the solution-grown single crystals with scotch tape and transferred onto the ITO substrates. The high-current regions in the cAFM images are related with the exposed ITO substrates. Both the $(Cl4Tm)_2PbI_4$ and $(Br4Tm)_2PbI_4$ have a thickness of around 30-35 nm. $(Cl4Tm)_2PbI_4$ crystal exhibits low current below the detection limit of the cAFM even at high voltage bias. The $(Br4Tm)_2PbI_4$ crystal shows various current within the crystal region, which could be due to the different thickness, defect densities and heterogeneity in crystal quality.

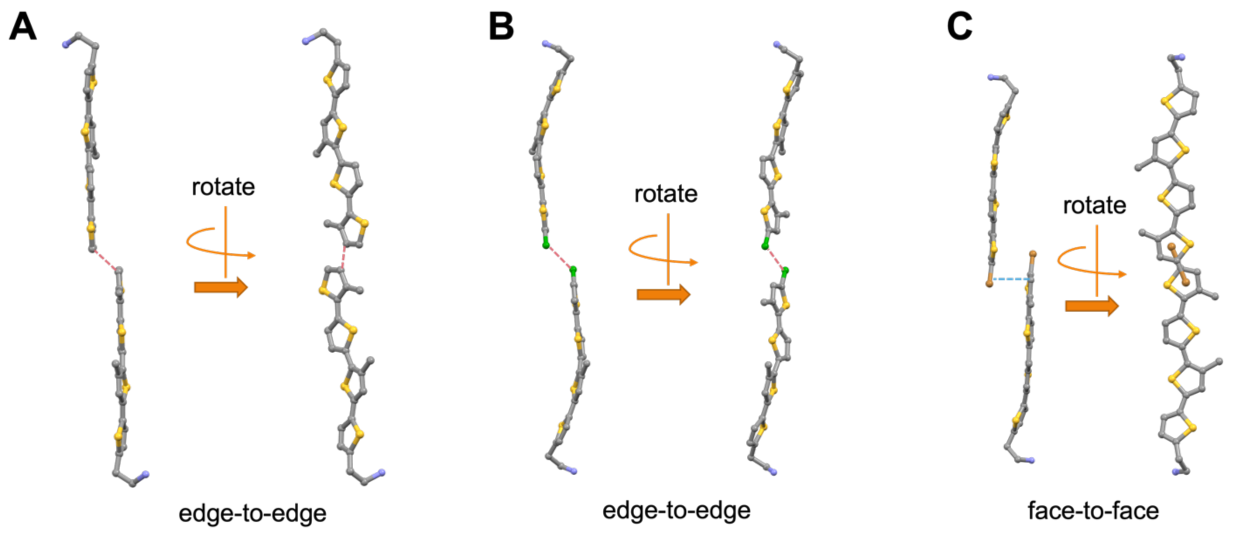

Fig. S7. The ligand packing geometry in crystal structures of layered perovskite. (**A**) $(4Tm)_2PbI_4$ (**B**) $(Cl4Tm)_2PbI_4$ and (**C**) $(Br4Tm)_2PbI_4$, from two different perspectives. Both $(4Tm)_2PbI_4$ and $(Cl4Tm)_2PbI_4$ show edge-to-edge ligand packing geometry on the outer-thiophene, while $(Br4Tm)_2PbI_4$ exhibit face-to-face ligand packing with Br atom close to the aromatic core of the outer-thiophene. The chloroform solvent molecules are far away from the center of face-to-face packing and would not affect the of Br4Tm interactions.

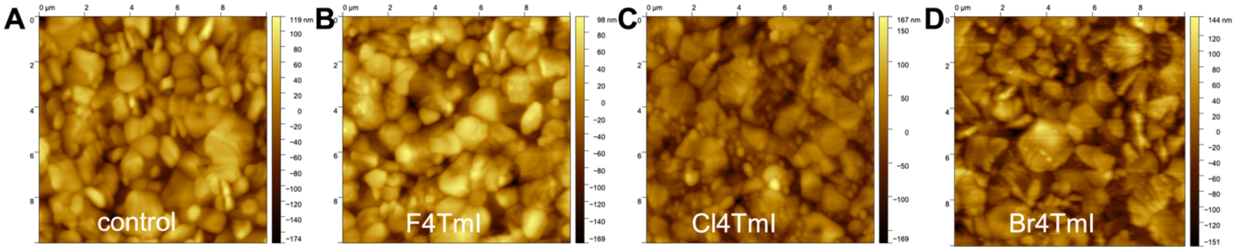

Fig. S8. AFM images of perovskite thin films. (**A**) Untreated perovskite surface and 2D/3D heterostructure formed with (**B**) F4TmI, (**C**) Cl4TmI, and (**D**) Br4TmI. No significant differences in surface morphology have been observed in these films.

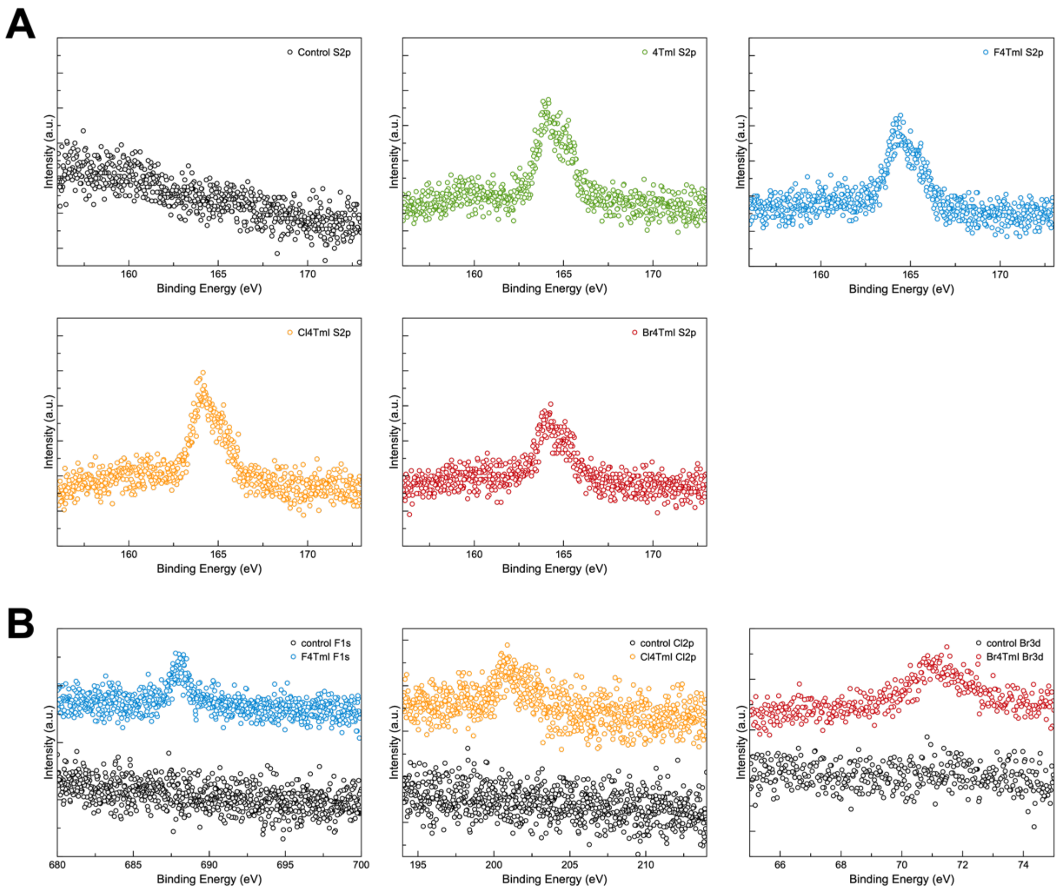

Fig. S9. XPS plots of perovskite thin films. (**A**) S 2p and (**B**) respective halogen regions on perovskite thin films with different surface treatments.

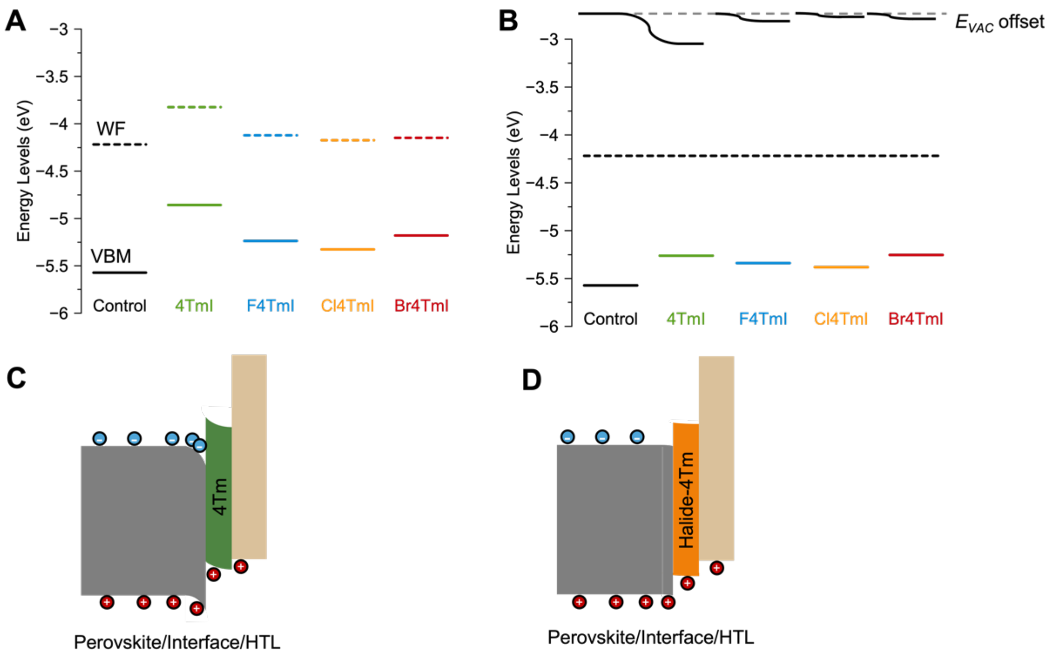

Fig. S10. Energy band diagrams extracted from UPS results. (**A**) The WF and VBM of thin films without and with surface treatment. The significant upshift of the WF and VBM of 4TmI comparing to other halogen-4TmI ligands are due to the shallower HOMO levels of 4TmI. (**B**) Vacuum level shift after band alignment. (**C**) Schematic illustration of interface band alignment and charge distribution when 4TmI is used for surface treatment. The green region indicates the surface of 3D perovskite treated with 4TmI ligand. In general, the surface of perovskite has upshifted VBM compared to the bulk 3D film. The interfacial energy landscape between 3D and 2D perovskite with 4TmI will likely increase recombination due to the accumulation of electrons at the 3D/2D interface, which is caused by the upshifted WF of 4TmI treated surface. (**D**) Schematic illustration of interface band alignment with small vacuum level shift when halogen-4TmI ligands are used for surface treatment. The surface of perovskite thin films still maintains upshifted VBM, while the unfavorable band bending at the interface between 3D and 2D perovskite is negligible due to the small change of WF of halogen-4TmI treatment. All the surface treatments generate type-II alignment at interface, which favors hole transfer from perovskite to HTL.

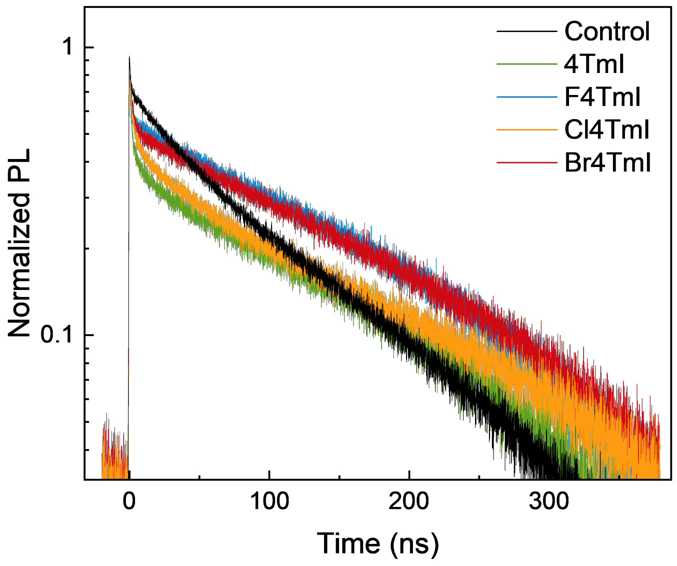

Fig. S11. Time-resolved photoluminescence (TRPL) of 3D perovskite thin films with different ligands treatments. All the samples are prepared on glass substrates. The control film exhibits almost mono-exponential decay with short lifetime. However, surface treated thin films all exhibit clear bi-exponential decay with a fast decay initially and a slow decay in the second stage. The fast decay occurs within 20 ns, which is usually correlated with the hole extraction effect of these conjugated ligands. The second stage of slow decay is usually related with trap-induced decay, while the longer lifetime of ligand treated films, comparing with control sample, indicates the reduced defect density.

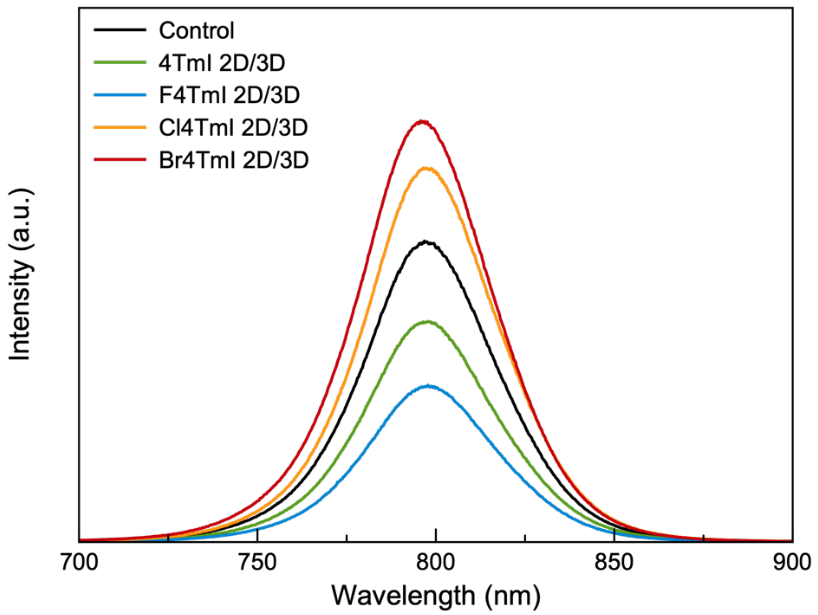

Fig. S12. Photoluminescence (PL) spectra of 3D perovskite thin films with different ligands treatments. All the samples are prepared on glass substrates. The increased PL intensity of Cl4TmI and Br4TmI is attributed to passivated defects and less trapped electrons. The decreased PL intensity of F4TmI is potentially due to the disordered surface energy resulted from forming unfavorable 2D structures. The decreased PL intensity of 4TmI is potentially due to the trapped electrons according to the energy alignment analysis.

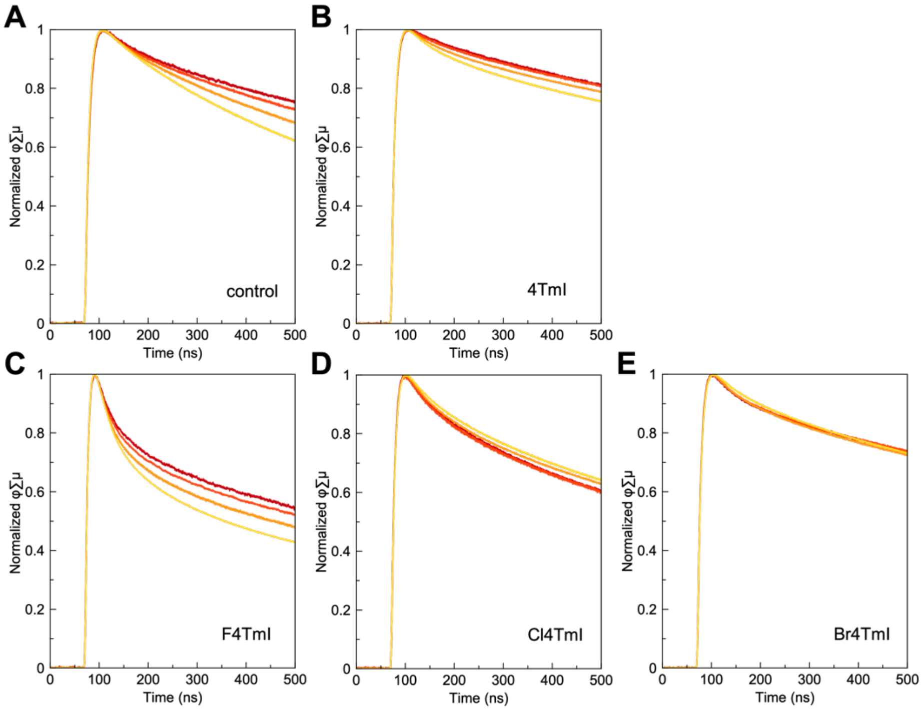

Fig. S13. Intensity-dependent photoconductivity transients of perovskite films with different surface treatments, measured by TRMC. The 650 nm (10 Hz, ca. 5 ns pulse width) excitation power decreases as the curves changes from dark red to light yellow. The samples with 4TmI, Cl4TmI and Br4TmI surface treatment exhibit obviously improved intensity-independent properties, comparing with control sample, which suggests decreased bimolecular recombination processes. However, F4TmI surface treatment worsens the free carrier recombination with the more intensity-dependence on the excitation power.

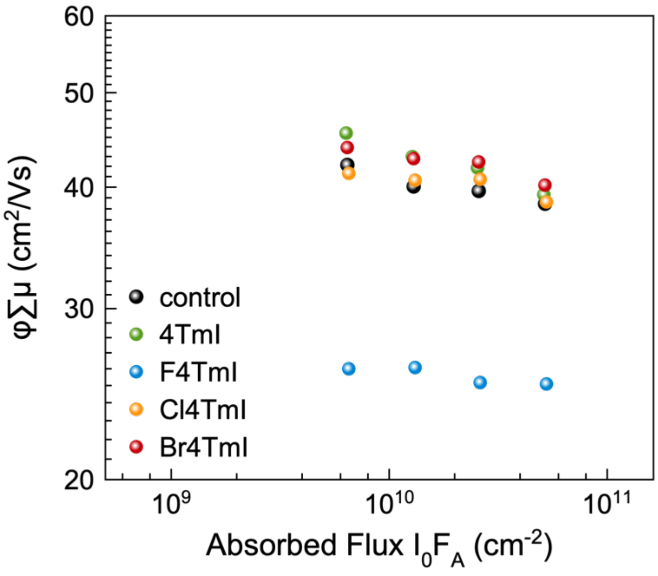

Fig. S14. TRMC comparison of carrier transport in 3D/2D perovskite thin films with different ligand treatment. The samples without or with 4TmI, Cl4TmI and Br4TmI treatment exhibit similar mobility, which is dominated by the carrier mobility in 3D perovskite due to the ultra-thin layer of 2D perovskite. However, the F4TmI surface treatment significantly deteriorates the mobility, which is consistent with the low mobility observed in n=1 2D perovskite films, and the high carrier recombination in F4TmI treated 3D perovskite films.

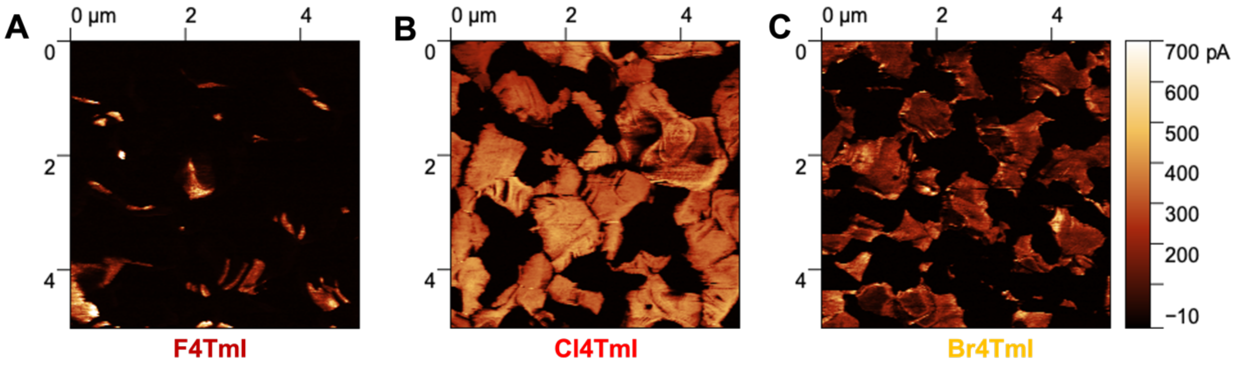

Fig. S15. cAFM images of 2D/3D heterostructure formed with different ligands. Since the 3D perovskite thin films were prepared with a two-step spin coating method, the unavoidable extra PbI_2 exists within the films, which contributes to the low conductivity regions in all the images. This is also observed in the pristine perovskite films without any surface treatment.

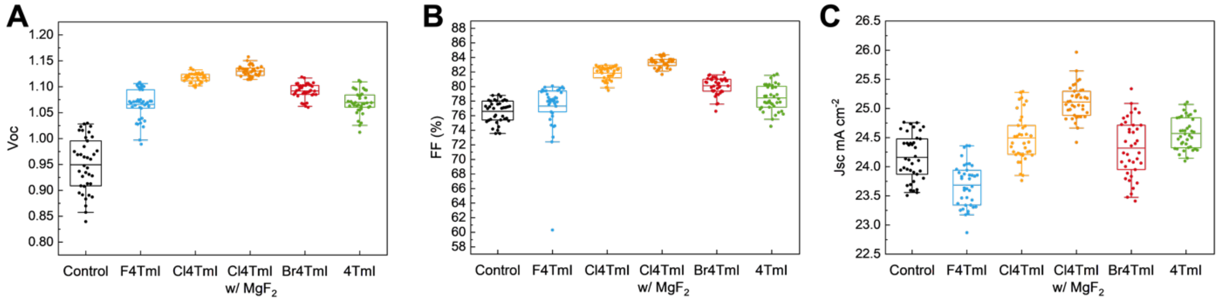

Fig. S16. Statistics of device parameters of PSCs without and with different ligand surface treatments. (**A**) V_{SC} (**B**) *FF* and (**C**) J_{SC}

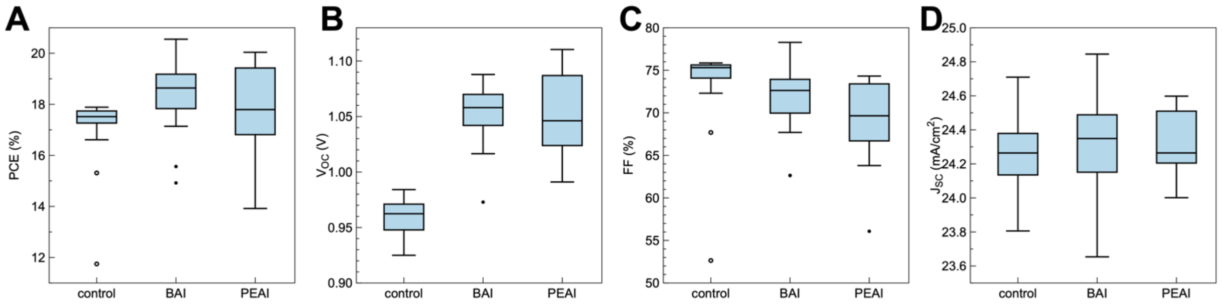

Fig. S17. Statistics of PSCs treated with PEAI and BAI, comparing to the control device without surface treatment. (**A**) PCE, (**B**) V_{OC}, (**C**) FF and (**D**) J_{SC}.

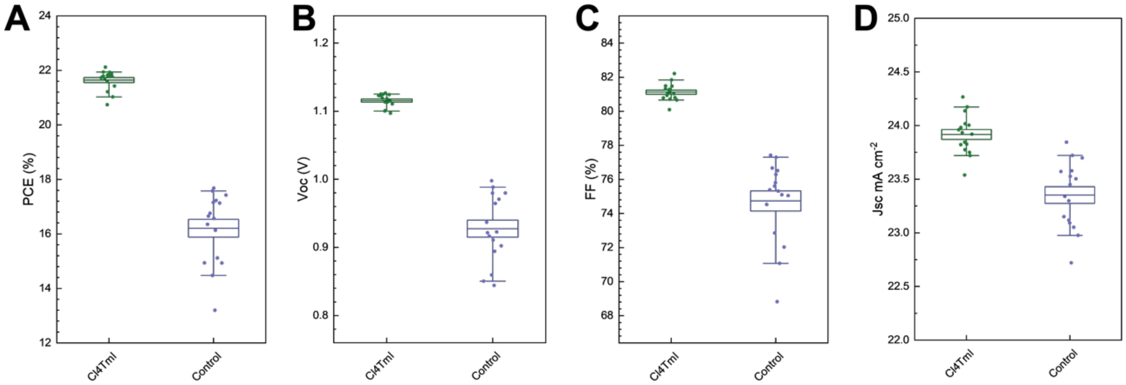

Fig. S18. Statistics of $FA_{0.88}MA_{0.07}Cs_{0.05}PbI_{2.89}Br_{0.11}$ devices with and without Cl4TmI surface treatment. (**A**) PCE, (**B**) V_{OC}, (**C**) FF and (**D**) J_{SC}.

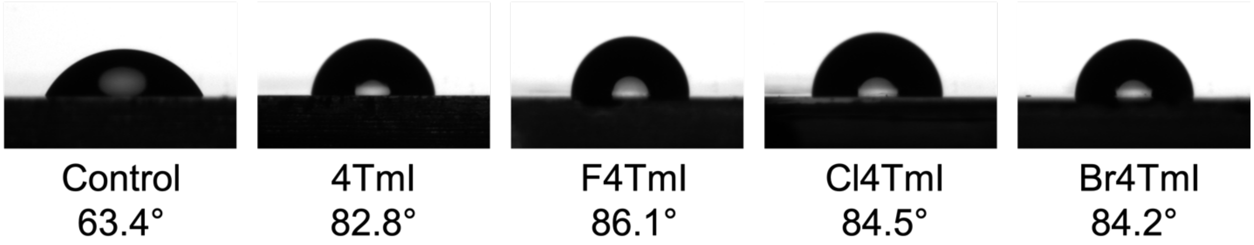

Fig. S19. Water contact angles of 3D perovskite thin films treated with different ligands. The labels indicate the water contact angles of the corresponding thin films. All the surface treated perovskite thin films exhibit significantly increased water contact angles. Among them, the halogen-4TmI samples show very similar water contact angles.

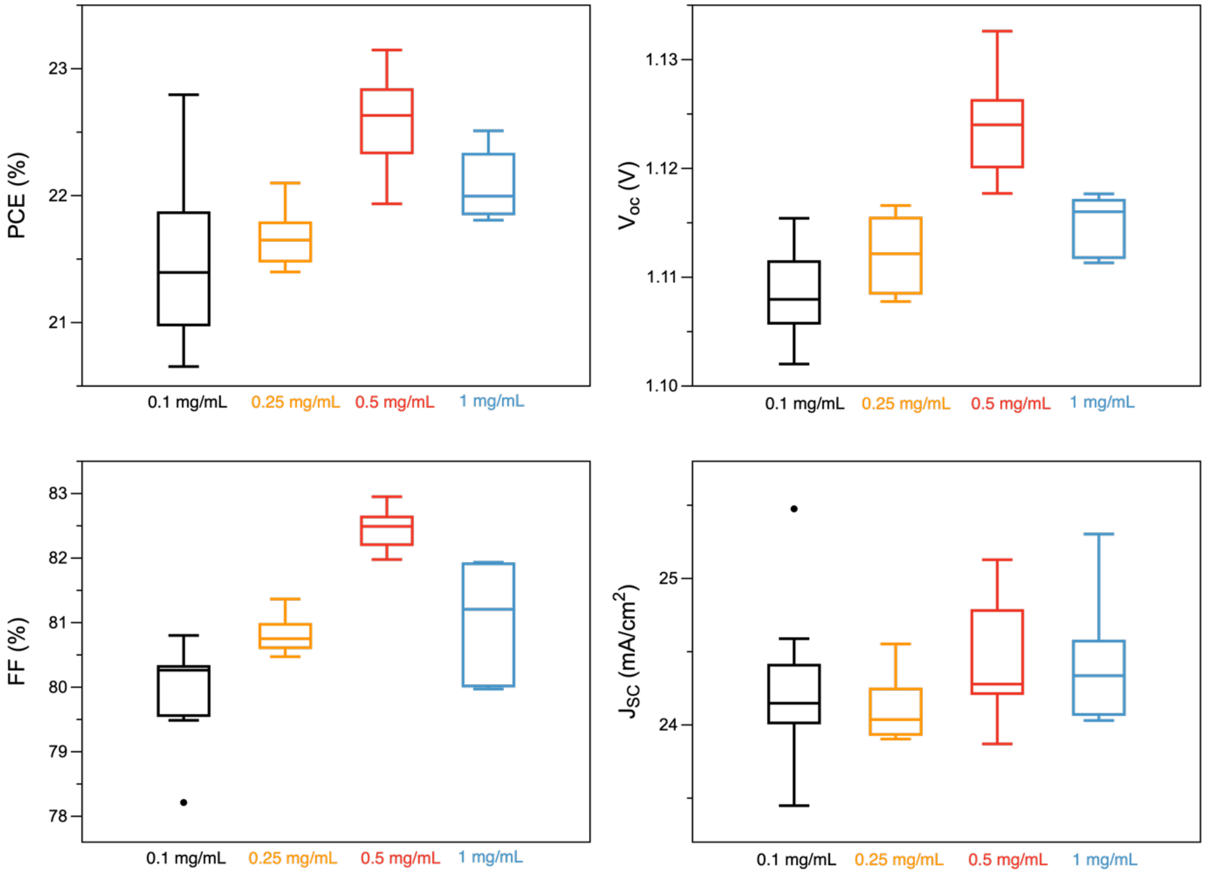

Fig. S20. Statistics of PCE, V_{OC}, FF and J_{SC} of PSCs with Cl4TmI treatment at different concentrations.

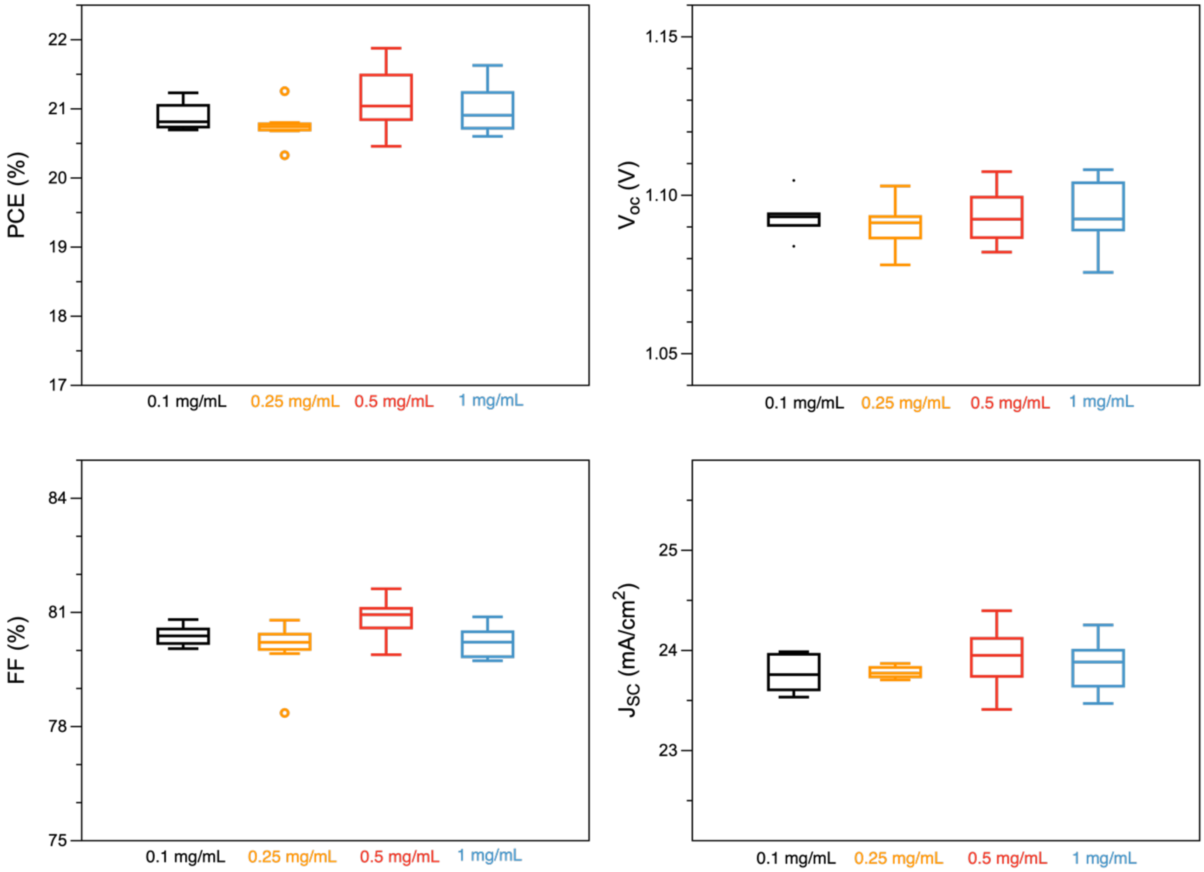

Fig. S21. Statistics of PCE, V_{OC}, FF and J_{SC} of PSCs with Br4TmI treatment at different concentrations.

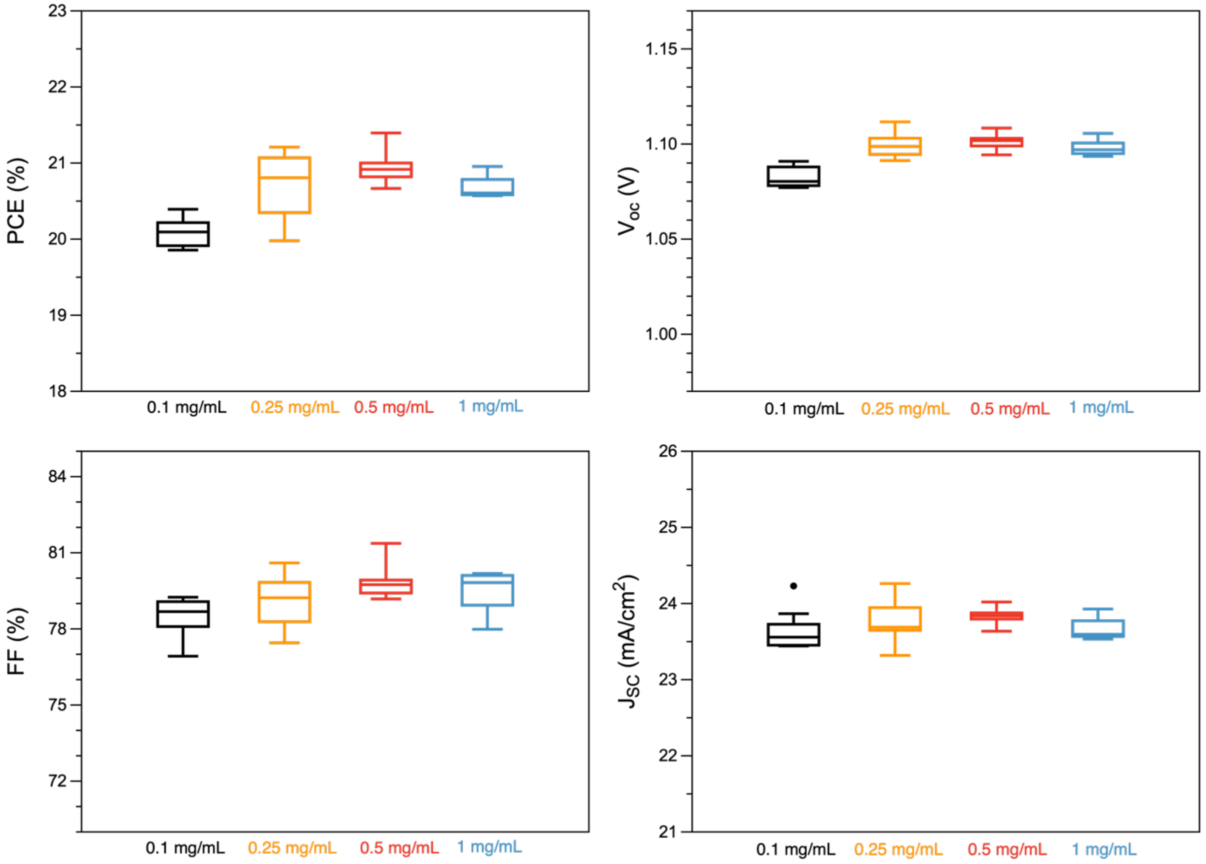

Fig. S22. Statistics of PCE, V_{OC}, *FF* and J_{SC} of PSCs with F4TmI treatment at different concentrations.

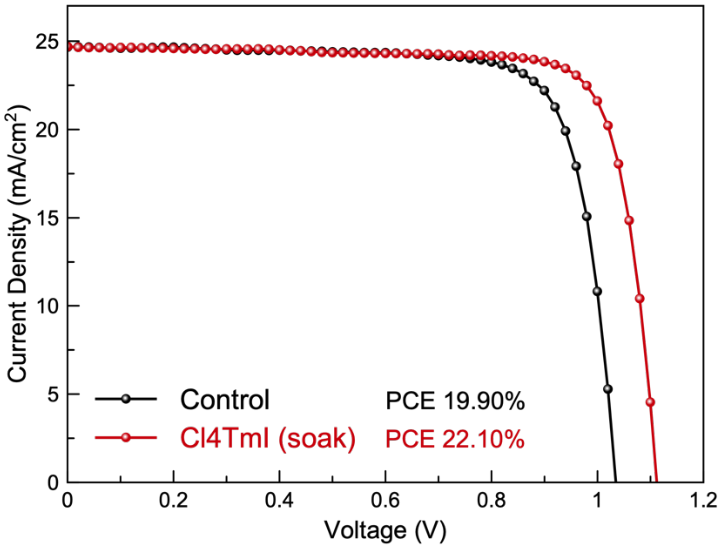

Fig. S23. ***J-V*** **characteristic of a control device and a device treated with Cl4TmI by allowing the ligand solution to stay on the surface of 3D thin films for extended time before spun off.** The method used to prepare the ligand-treated device was used to prepare XRD samples. The result demonstrated that even with the extended reaction time between ligand and the 3D perovskite, the treatment effect still can be preserved.

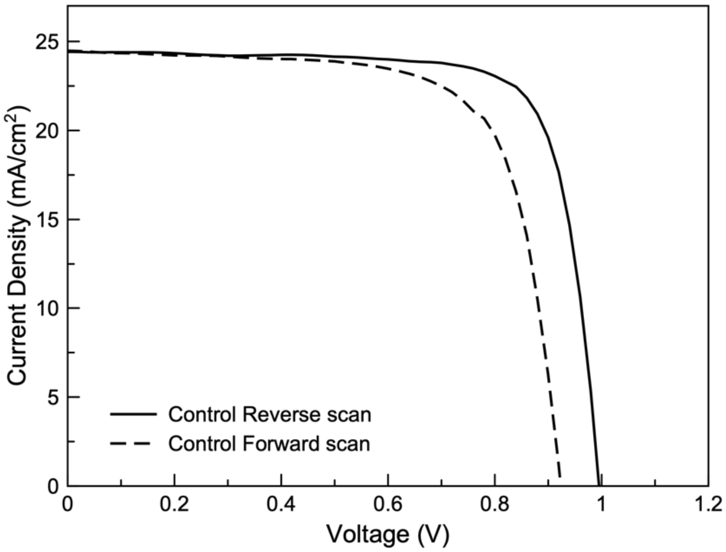

Fig. S24. ***J-V*** **characteristic of a typical control device with reverse and forward scans.**

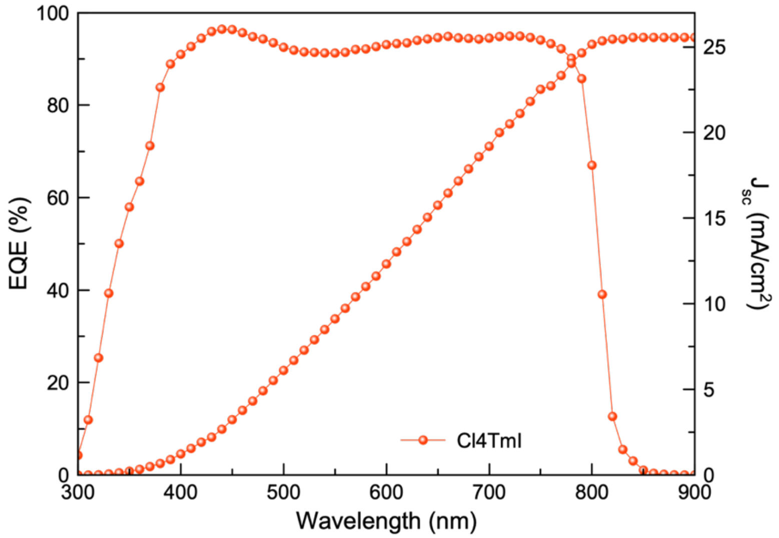

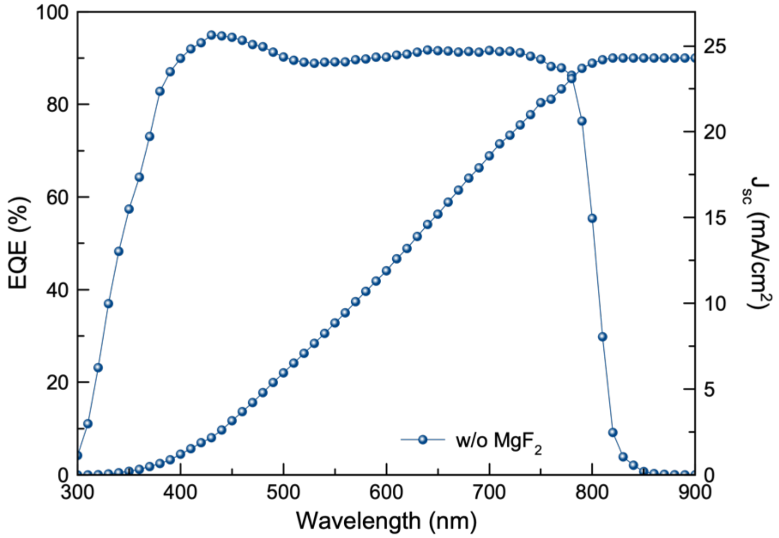

Fig. S25. The external quantum efficiency (EQE) spectra of devices with Cl4TmI treatment. Top: EQE of a champion device, together with the integrated J_{sc} calculated from the EQE spectrum. Bottom: EQE and integrated J_{sc} of a Cl4TmI-treated device without MgF_2 anti-reflection coating.

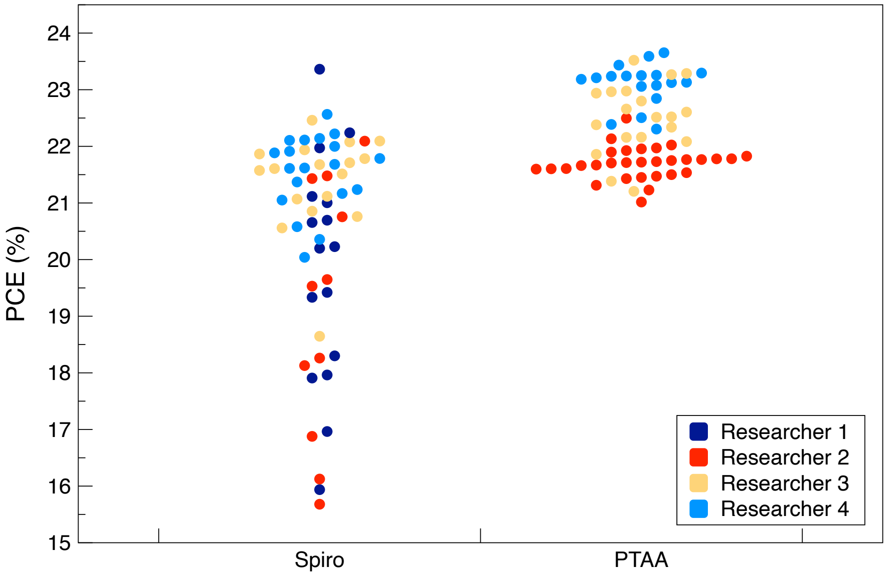

Fig. S26. Statistics of PCEs of PSCs fabricated with different researchers to confirm the reproducibility of our strategy (denoted as Researcher 1 to Researcher 4). One of the researchers is an undergraduate student. The large variation in Spiro-based devices is caused by different air-aging conditions, including humidity and temperature, and the dopant solution storage time.

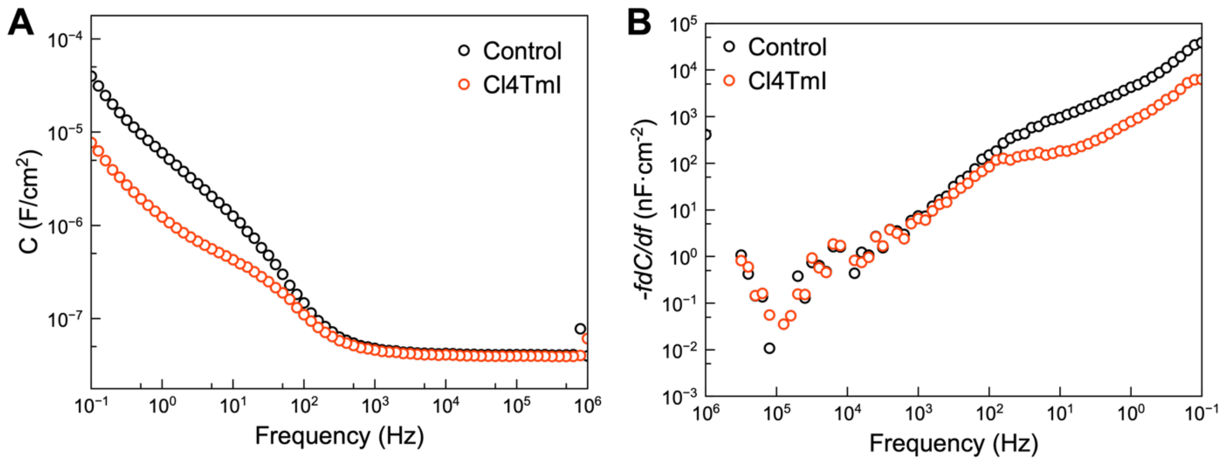

Fig. S27. The electrochemical characterization of the PSC devices without and with Cl4TmI treatment to indicate ion migration and defect density. (**A**) Capacitance-frequency (*C-f*) profile and (**B**) *-f·dC/df - f* plots of control and Cl4TmI-treated devices are measured at room temperature. The lower capacitance in C-f profiles of Cl4TmI at low frequency region indicates fewer mobile ions. The lower value of *-f·dC/df* of Cl4TmI sample indicates the lower defect density within the corresponding frequency region.

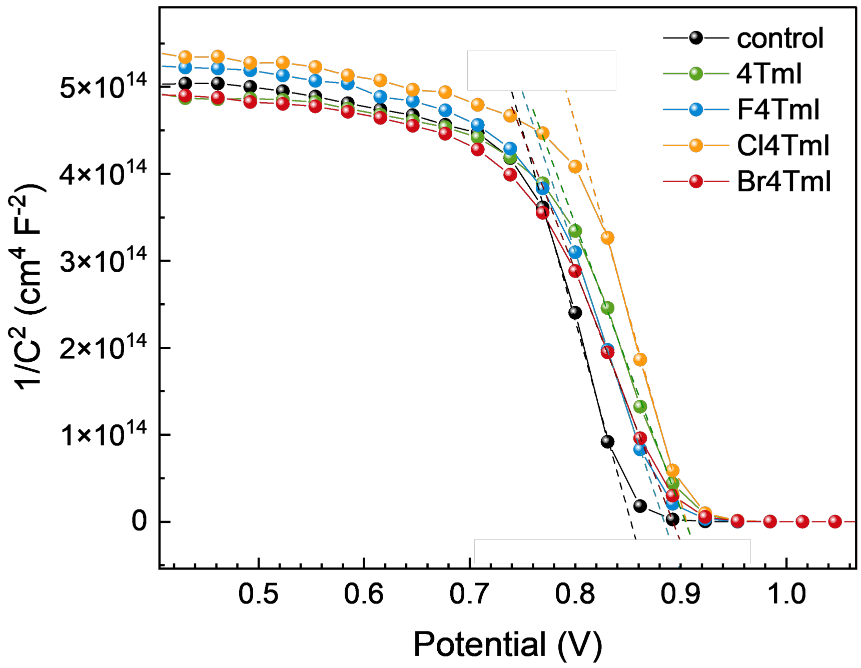

Fig. S28. Mott-Schottky plots of devices with different ligand treatment.

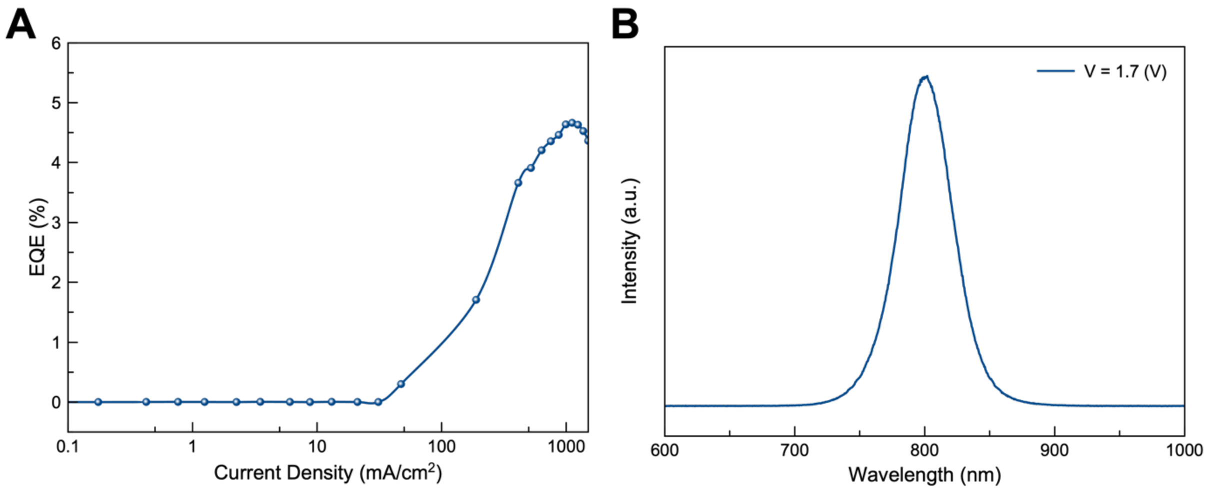

Fig. S29. Electroluminescence (EL) characterization. (**A**) EQE of EL of the devices while operating as LEDs. (**B**) EL spectra of the devices with Cl4TmI treatment.

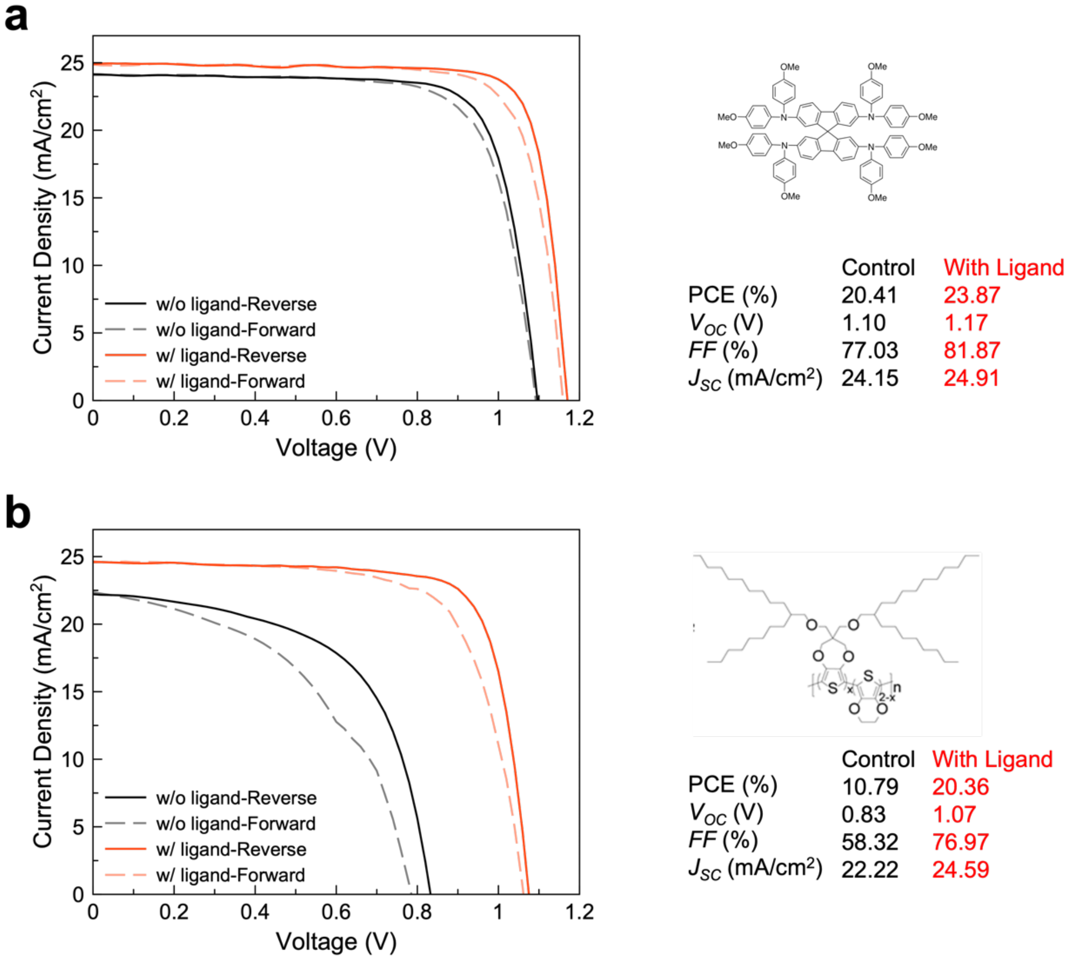

Fig. S30. ***J-V*** **characteristics of PSCs fabricated with different hole transporting materials, comparing the device without and with surface treatment.** (**A**) Spiro-OMeTAD and (**B**) PE_2 devices. All the devices show both reverse and forward scans. PE_2 denotes the copolymers containing 3,4-ethylenedioxythiophene (EDOT) and 3,4-propylenedioxythiophene (ProDOT). The ligand used in Spiro-OMeTAD and PE_2 devices is Cl4TmI.

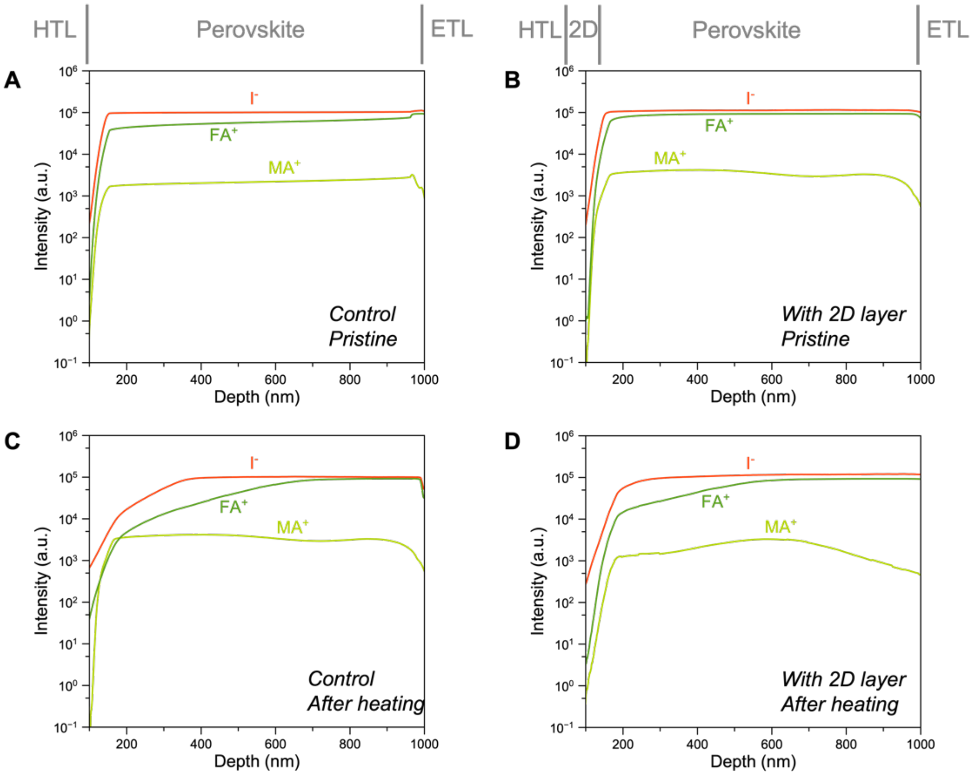

Fig. S31. The depth profiles of ToF-SIM measurements of devices after thermal treatment. The ToF-SIM measurements of control devices (**A** and **C**) and Cl4TmI-treated devices (**B** and **D**). (**A** and **B**) Devices without thermal treatment. (**C** and **D**) Devices after 85 °C treatment for 120 hr as accelerated thermal stability test. The iodide showed obvious migration in control devices after thermal treatment, while the iodide in 2D-treated devices showed much insignificant distribution change, which indicate the 2D treatment can effectively slow down the interface ion migration.

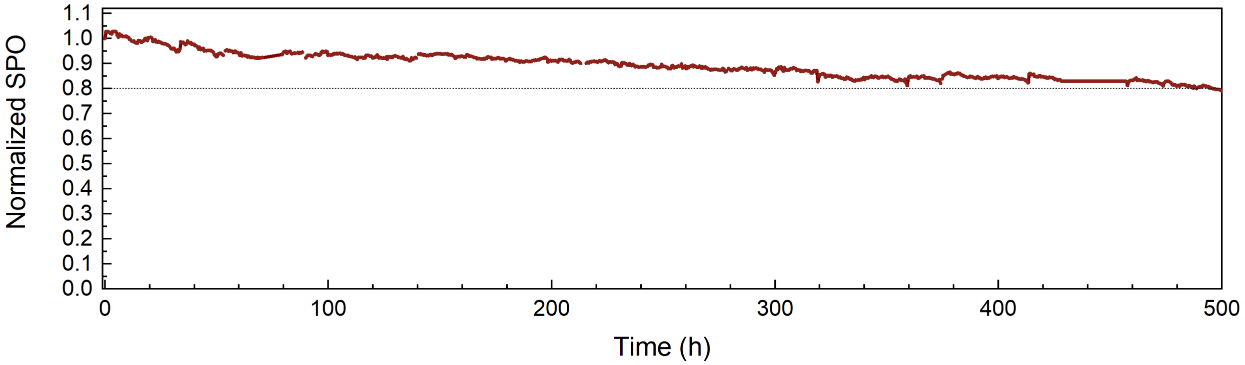

Fig. S32. Stability tracking under maximum power output of Cl4TmI treated device. The measurement was performed in the N_2 filled glovebox without encapsulation. The initial PCE of the measured device is 20.8%.

3. Supplementary tables and discussion

Table S1. Cyclic voltammetry analysis of different ligands.

	4TmI	F4TmI	Cl4TmI	Br4TmI
$E_{on\text{-}set}$ vs. reference V	0.83	0.87	0.88	0.87
$E_{on\text{-}set}$ (vacuum) eV	-5.21	-5.25	-5.26	-5.25
$E_{1/2}$ vs. reference V	0.92	0.95	0.96	0.96
$E_{1/2}$ (vacuum) eV	-5.19	-5.21	-5.22	-5.22

The calculation is based on the ferrocene calibration. The standard redox potential of Fc^{+}/Fc^{0} is −4.8 eV vs. vacuum. We measured $Fc^{+}/Fc^{0}{}_{onset}$ = 0.42 V vs. Ag/AgCl reference electrode, and $Fc^{+}/Fc^{0}{}_{1/2}$ = 0.54 V vs. Ag/AgCl reference electrode. Therefore, $Ag/AgCl_{on\text{-}set}$ is calibrated as −4.38 eV vs. vacuum, and $Ag/AgCl_{1/2}$ is calibrated as −4.26 eV vs. vacuum. The HOMO level (vacuum) of each ligand is calculated based on equations shown below:

$$E_{on\text{-}set}\text{ (vacuum) eV} = -E\text{ (vs. reference)} - 4.38\text{ eV}$$

$$E_{1/2}\text{ (vacuum) eV} = -E\text{ (vs. reference)} - 4.26\text{ eV}$$

Table S2. **Energy levels of perovskite films extracted from UPS results.** The E_{VAC} offset indicate the shift of the WF from the control sample after ligand treatment. The aligned VBM is calculated based on the aligned Fermi levels.

	**WF (eV)**	**IE (eV)**	**E_{VAC} offset (eV)**	**Aligned VBM (eV)**
Control	4.22	5.57	-	-5.57
4TmI	3.82	4.86	-0.4	-5.26
F4TmI	4.12	5.24	-0.1	-5.34
Cl4TmI	4.17	5.33	-0.05	-5.38
Br4TmI	4.15	5.18	-0.07	-5.25

The significant difference of IE between 4TmI and halogen-4TmI modified perovskite surfaces is a combined effects of charge extraction efficiency of different ligands, the interactions between Pb-I framework and the ligand molecules, and the packing geometry of ligands on perovskite surface which is affected by halogen interactions.

Table S3. **Space-charge-limited current (SCLC) analysis of devices prepared with different surface treatment.** The V_{TFL} extrapolated from SCLC plots, while the hole mobility were calculated from fitting the Child region in SCLC plots.

	V_{TFL} (V)	Trap density (cm^{-3})	Hole mobility ($cm^2s^{-1}V^{-1}$)
Control	0.307	6.6279 x 10^{14}	0.086317 x 10^{-2}
4TmI	0.198	4.4274x 10^{14}	5.579147 x 10^{-2}
F4TmI	0.147	3.1736 x 10^{14}	5.810051 x 10^{-2}
Cl4TmI	0.13	2.8066 x 10^{14}	6.923628 x 10^{-2}
Br4TmI	0.102	2.2021 x 10^{14}	7.166453 x 10^{-2}

Table S4. **TRPL fitting results of ligand treated 3D perovskites with PTAA hole transport layer covering on the top.**

	τ_1 (ns)	A_1	τ_2 (ns)	A_2
Control	0.71	0.70	9.34	0.23
4TmI	0.48	0.83	10.54	0.14
F4TmI	0.64	0.75	13.56	0.19
Cl4TmI	0.60	0.70	20.65	0.20
Br4TmI	0.53	0.76	11.83	0.17

Table S5. The built-in potential（V_{bi}) calculated from Mott-Schottky plots.

	V_{bi}
control	0.853
4TmI	0.904
F4TmI	0.884
Cl4TmI	0.905
Br4TmI	0.893

Table S6. Single crystal X-ray diffraction data of $(Cl4Tm)_2PbI_4$

	$(Cl4Tm)_2PbI_2$
Crystal data	
Chemical formula	$I_8Pb_2 \cdot 4(C_{20}H_{19}ClNS_4)$
M_r	3177.78
Crystal system, space group	Triclinic, P -1
Temperature (K)	150
a, *b*, *c* (Å)	12.2750 (16), 12.3224 (17), 33.363 (5)
α, β, γ (°)	97.679 (9), 92.458 (7), 90.964 (11)
V (Å^3)	4995.2 (12)
Z	2
Radiation type	Cu $K\alpha$
μ (mm^{-1})	30.33
Crystal size (mm)	0.09 × 0.08 × 0.02
Data collection	
Diffractometer	Bruker AXS D8 Quest diffractometer with PhotonIII_C14 charge-integrating and photon counting pixel array detector
Absorption correction	Multi-scan *SADABS* 2016/2: Krause, L., Herbst-Irmer, R., Sheldrick G.M. & Stalke D. (2015). J. Appl. Cryst. 48, 3-10.
T_{min}, T_{max}	0.027, 0.144
No. of measured, independent and observed [I > 2σ(I)] reflections	50785, 50785, 36756
R_{int}	0.093
$(\sin \theta/\lambda)_{max}$ (Å^{-1})	0.638
Refinement	
$R[F^2 > 2\sigma(F^2)]$, $wR(F^2)$, S	0.100, 0.316, 1.07
No. of reflections	50785
No. of parameters	1476
No. of restraints	3034
H-atom treatment	H-atom parameters constrained
$\Delta\rho_{max}$, $\Delta\rho_{min}$ (e Å^{-3})	5.29, −3.92

Computer programs: Data reduction and correction was performed with Apex4 v2022.1-1 (2022) and *SAINT* V8.40B (2020) software. The crystal structure was solved and refined with SHELXT, *SHELXL2018*/3 and SHELXLE Rev1385 software (*40-43*).

Description for $(Cl4Tm)_2PbI_4$

The structure suffers from several types of non-merohedral twinning, leading to near to excessive overlap of a large fraction of reflections, preventing simultaneous integration of twin domains.
The main type of twinning was found to be a rotation around the reciprocal (0 0 1) axis (twin transformation matrix -1 0 0, 0 -1 0, 0.245 0.727 1). Multiple other types of twinning appear to be present, but were not well enough resolved to be unambiguously assigned. Attempts of simultaneous integration of the two main twin domains (related by the rotation around the reciprocal (0 0 1) axis proofed problematic due to excessive multiple overlap of reflections, resulting in large numbers of rejected reflections.
Attempts were made to adjust integration parameters to avoid excessive rejections (through adjustments to integration queue size, integration box slicing and twin overlap parameters, and omission of box size optimization), which led to less but still substantial numbers of rejected reflections. Attempts at refinement against these incomplete and biased data gave unsatisfactory results (R1 > 15%, largest difference peaks > 8 and < -5.9 electrons /Angstrom cubed).
With no complete data set obtainable through simultaneous integration of both twin domains, the data were instead handled as if not twinned, with only the major domain integrated, and converted into an hklf 5 type format hkl file after integration using the "Make HKLF5 File" routine as implemented in WinGX. The twin law matrix was used as obtained from SAINT, see above. The Overlap R1 and R2 values used were 0.45, i.e. reflections with a discriminator function less or equal to overlap radius of 0.45 were counted overlapped, all others as single. The discriminator function used was the "delta function on index non-integrality". No reflections were omitted.
The structure was solved using dual methods (ShelXT) with the original hklf 4 type file and was refined using the hklf 5 type file created via WinGX, resulting in a BASF value of 0.333(2). Omission of twinning results in apparent disorder for the entire PbI3 layer with alternative Pb positions at the center of each PbI4 square, and apparent terminal I atoms at the positions of the ammonium H3N-CH2 units (Q peaks up to 8.01 electrons / Angstrom cubed, double the values for the largest difference densities with twinning included). No Rint value is obtainable for the hklf 5 type file using the WinGX routine. The value from the original HKLF 4 type file is given

instead. The structure exhibits pseudo-translations along the a- and b-axes. Exact translational symmetry is broken by modulation of the iodine atoms and torsion angles of the ammonium H_3N-CH_2 units. The remainder of the cations exhibits close to perfect translational symmetry along b (but not a).

Minor disorder of the PbI_3 layer is observed, with Pb ions located at both possible positions above and below the average Pb-I plane (being bonded to either the top or bottom terminal iodine atom), and with bridging layer iodine atoms disordered by a pseudo-glide operation along either a or b (or a ca. 25 degree rotation around the I-Pb-I axis involving the terminal iodines). The Pb and I disorder refined to similar but slightly different occupancies. ADPs of major and minor equivalent Pb and I atoms were constrained to be identical. Subject to these conditions the occupancy ratios refined to 0.969(2) to 0.031(2) for the lead ions, and to 0.9576(19) to 0.0424(19) for the iodine atoms.

Disorder is observed for the cations by rotation of the outermost ring. The disorder extends to the directly adjacent thiophene. All cation moieties were restrained to have similar geometries (bond distances and angles, but not torsion angles, which differ for rings 1 and 2 for residues 1 and 2 versus 3 and 4). All C-C(methyl) bond distances were restrained to be similar to each other. Uij components of ADPs for disordered atoms closer to each other than 2.0 Angstrom were restrained to be similar. Major and minor moieties of residues 1 and 2, and residues 3 and 4 are each mutually incompatible (due to close contacts of Cl atoms across layers), and common occupancy ratios were used. For molecules 1 and 2 occupancies refined to close to 1:1, for molecules 3 and 4 one of the disordered moieties is each dominant. For the minor moieties, the outer rings were restrained to be close to planar. For residue 3, the adjacent ring was also restrained to be close to planar, and atom C12 was omitted from the disorder. For residue 4 atoms C12 and C12B were constrained to have identical ADPs. The bond distances C10 to C12 and C10 to C12B were restrained to be similar for all cations. Subject to these conditions the occupancy ratios refined to 0.517(12) to 0.483(12) for residue 1 and 2, and to 0.885(15) to 0.115(15) for residues 3 and 4.

Table S7. Single crystal X-ray diffraction data of $(Br4Tm)_2PbI_4$

	$(Br4Tm)_2PbI_4$
Crystal data	
Chemical formula	$I_8Pb_2\cdot4(C_{20}H_{19}BrNS_4)\cdot2(CHCl_3)$
M_r	3594.36
Crystal system, space group	Triclinic, *P*-1
Temperature (K)	150
a, *b*, *c* (Å)	12.029 (2), 12.3491 (17), 36.860 (7)
α, β, γ (°)	80.804 (9), 86.699 (8), 89.799 (10)
V (Å^3)	5396.2 (16)
Z	2
Radiation type	Cu $K\alpha$
μ (mm^{-1})	30.30
Crystal size (mm)	0.07 × 0.05 × 0.01
Data collection	
Diffractometer	Bruker AXS D8 Quest
Absorption correction	Multi-scan<br>*SADABS* 2016/2: Krause, L., Herbst-Irmer, R., Sheldrick G.M. & Stalke D., J. Appl. Cryst. 48 (2015) 3-10
T_{min}, T_{max}	0.053, 0.178
No. of measured, independent and observed [$I > 2\sigma(I)$] reflections	64866, 21600, 13748
R_{int}	0.110
$(\sin\theta/\lambda)_{max}$ (Å^{-1})	0.637
Refinement	
$R[F^2 > 2\sigma(F^2)]$, $wR(F^2)$, S	0.066, 0.192, 1.03
No. of reflections	21600
No. of parameters	1120
H-atom treatment	H-atom parameters constrained
	$w = 1/[\sigma^2(F_o^2) + (0.0537P)^2 + 14.3139P]$<br>where $P = (F_o^2 + 2F_c^2)/3$
$\Delta\rho_{max}$, $\Delta\rho_{min}$ (e Å^{-3})	2.58, −1.61

Computer programs: Data reduction and correction was performed with Apex4 v2022.1-1 (2022) and *SAINT* V8.40B (2020) software. The crystal structure was solved and refined with SHELXT, *SHELXL2018*/3 and SHELXLE Rev1385 software (*40-43*).

Description for $(Br4Tm)_2PbI_4$

The structure metrically emulates a monoclinic C-centered lattice, but is not significantly twinned by that symmetry. Application of the twin transformation matrix -1 0 0 0 1 0 0 1 -1 yielded a BASF value of only 2.71(9)%. The lead ions were refined as disordered over the two displaced positions inside the iodine octahedra. ADPs of equivalent lead ions were constrained to be identical. Subject to these conditions the occupancy ratios refined to 0.9896(17) to 0.0104(17) (Pb1) and 0.983(3) to 0.017(3) (Pb2)

REFERENCES AND NOTES

1. M. Grätzel, The rise of highly efficient and stable perovskite solar cells. *Acc. Chem. Res.* **50**, 487–491 (2017).

2. H. Min, D. Y. Lee, J. Kim, G. Kim, K. S. Lee, J. Kim, M. J. Paik, Y. K. Kim, K. S. Kim, M. G. Kim, T. J. Shin, S. I. Seok, Perovskite solar cells with atomically coherent interlayers on SnO_2 electrodes. *Nature* **598**, 444–450 (2021).

3. Y. Zhao, Z. Qu, S. Yu, T. Shen, H. Deng, X. Chu, X. Peng, Y. Yuan, X. Zhang, J. You, Inactive $(PbI_2)_2RbCl$ stabilizes perovskite films for efficient solar cells. *Science* **377**, 531–534 (2022).

4. S. Tan, T. Huang, I. Yavuz, R. Wang, T. W. Yoon, M. Xu, Q. Xing, K. Park, D.-K. Lee, C.-H. Chen, R. Zheng, T. Yoon, Y. Zhao, H.-C. Wang, D. Meng, J. Xue, Y. J. Song, X. Pan, N.-G. Park, J.-W. Lee, Y. Yang, Stability-limiting heterointerfaces of perovskite photovoltaics. *Nature* **605**, 268–273 (2022).

5. Y. Wang, I. Ahmad, T. Leung, J. Lin, W. Chen, F. Liu, A. M. C. Ng, Y. Zhang, A. B. Djurišić, Encapsulation and stability testing of perovskite solar cells for real life applications. *ACS Mater. Au.* **2**, 215–236 (2022).

6. R. Wang, M. Mujahid, Y. Duan, Z. K. Wang, J. Xue, Y. Yang, A review of perovskites solar cell stability. *Adv. Funct. Mater.* **29**, 1808843 (2019).

7. Q. Jiang, J. Tong, Y. Xian, R. A. Kerner, S. P. Dunfield, C. Xiao, R. A. Scheidt, D. Kuciauskas, X. Wang, M. P. Hautzinger, R. Tirawat, M. C. Beard, D. P. Fenning, J. J. Berry, B. W. Larson, Y. Yan, K. Zhu, Surface reaction for efficient and stable inverted perovskite solar cells. *Nature* **611**, 278–283 (2022).

8. Q. Jiang, Y. Zhao, X. Zhang, X. Yang, Y. Chen, Z. Chu, Q. Ye, X. Li, Z. Yin, J. You, Surface passivation of perovskite film for efficient solar cells. *Nat. Photonics* **13**, 460–466 (2019).

9. Z. Wang, Q. Lin, F. P. Chmiel, N. Sakai, L. M. Herz, H. J. Snaith, Efficient ambient-air-stable solar cells with 2D–3D heterostructured butylammonium-caesium-formamidinium lead halide perovskites. *Nat. Energy* **2**, 17135 (2017).

10. Y.-W. Jang, S. Lee, K. M. Yeom, K. Jeong, K. Choi, M. Choi, J. H. Noh, Intact 2D/3D halide junction perovskite solar cells via solid-phase in-plane growth. *Nat. Energy* **6**, 63–71 (2021).

11. F. Zhang, H. Lu, J. Tong, J. J. Berry, M. C. Beard, K. Zhu, Advances in two-dimensional organic-inorganic hybrid perovskites. *Energ. Environ. Sci.* **13**, 1154–1186 (2020).

12. J. J. Yoo, G. Seo, M. R. Chua, T. G. Park, Y. Lu, F. Rotermund, Y.-K. Kim, C. S. Moon, N. J. Jeon, J.-P. Correa-Baena, V. Bulović, S. S. Shin, M. G. Bawendi, J. Seo, Efficient perovskite solar cells via improved carrier management. *Nature* **590**, 587–593 (2021).

13. F. Zhang, S. Y. Park, C. Yao, H. Lu, S. P. Dunfield, C. Xiao, S. Uličná, X. Zhao, L. D. Hill, X. Chen, X. Wang, L. E. Mundt, K. H. Stone, L. T. Schelhas, G. Teeter, S. Parkin, E. L. Ratcliff, Y.-L. Loo, J. J. Berry, M. C. Beard, Y. Yan, B. W. Larson, K. Zhu, Metastable Dion-Jacobson 2D structure enables efficient and stable perovskite solar cells. *Science* **375**, 71 (2022), 76.

14. S. Sidhik, Y. Wang, M. De Siena, R. Asadpour, A. J. Torma, T. Terlier, K. Ho, W. Li, A. B. Puthirath, X. Shuai, A. Agrawal, B. Traore, M. Jones, R. Giridharagopal, P. M. Ajayan, J. Strzalka, D. S. Ginger, C. Katan, M. A. Alam, J. Even, M. G. Kanatzidis, A. D. Mohite, Deterministic fabrication of 3D/2D perovskite bilayer stacks for durable and efficient solar cells. *Science* **377**, 1425–1430 (2022).

15. H. Chen, S. Teale, B. Chen, Y. Hou, L. Grater, T. Zhu, K. Bertens, S. M. Park, H. R. Atapattu, Y. Gao, M. Wei, A. K. Johnston, Q. Zhou, K. Xu, D. Yu, C. Han, T. Cui, E. H. Jung, C. Zhou, W. Zhou, A. H. Proppe, S. Hoogland, F. Laquai, T. Filleter, K. R. Graham, Z. Ning, E. H. Sargent, Quantum-size-tuned heterostructures enable efficient and stable inverted perovskite solar cells. *Nat. Photonics* **16**, 352–358 (2022).

16. R. Azmi, E. Ugur, A. Seitkhan, F. Aljamaan, A. S. Subbiah, J. Liu, G. T. Harrison, M. I. Nugraha, M. K. Eswaran, M. Babics, Y. Chen, F. Xu, T. G. Allen, A. U. Rehman, C.-L. Wang, T. D. Anthopoulos, U. Schwingenschlögl, M. De Bastiani, E. Aydin, S. De Wolf, Damp heat–stable perovskite solar cells with tailored-dimensionality 2D/3D heterojunctions. *Science* **376**, 73–77 (2022).

17. J. Xue, R. Wang, X. Chen, C. Yao, X. Jin, K.-L. Wang, W. Huang, T. Huang, Y. Zhao, Y. Zhai, D. Meng, S. Tan, R. Liu, Z.-K. Wang, C. Zhu, K. Zhu, M. C. Beard, Y. Yan, Y. Yang, Reconfiguring the band-edge states of photovoltaic perovskites by conjugated organic cations. *Science* **371**, 636–640 (2021).

18. Y. Gao, E. Shi, S. Deng, S. B. Shiring, J. M. Snaider, C. Liang, B. Yuan, R. Song, S. M. Janke, A. Liebman-Peláez, P. Yoo, M. Zeller, B. W. Boudouris, P. Liao, C. Zhu, V. Blum, Y. Yu, B. M. Savoie, L. Huang, L. Dou, Molecular engineering of organic–inorganic hybrid perovskites quantum wells. *Nat. Chem.* **11**, 1151–1157 (2019).

19. H. Tsai, R. Asadpour, J.-C. Blancon, C. C. Stoumpos, J. Even, P. M. Ajayan, M. G. Kanatzidis, M. A. Alam, A. D. Mohite, W. Nie, Design principles for electronic charge transport in solution-processed vertically stacked 2D perovskite quantum wells. *Nat. Commun.* **9**, 2130 (2018).

20. T. He, S. Li, Y. Jiang, C. Qin, M. Cui, L. Qiao, H. Xu, J. Yang, R. Long, H. Wang, M. Yuan, Reduced-dimensional perovskite photovoltaics with homogeneous energy landscape. *Nat. Commun.* **11**, 1672 (2020).

21. K. Ma, H. R. Atapattu, Q. Zhao, Y. Gao, B. P. Finkenauer, K. Wang, K. Chen, S. M. Park, A. H. Coffey, C. Zhu, L. Huang, K. R. Graham, J. Mei, L. Dou, Multifunctional conjugated ligand engineering for stable and efficient perovskite solar cells. *Adv. Mater.* **33**, 2100791 (2021).

22. A. Bala, V. Kumar, Effects of Cl and F substitution in phenylethylammonium spacer cations on stability, structure, and optical properties of 2D–3D ruddlesden–Popper perovskite layers. *ACS Appl. Energy Mater.* **4**, 1860–1867 (2021).

23. J. V. Passarelli, D. J. Fairfield, N. A. Sather, M. P. Hendricks, H. Sai, C. L. Stern, S. I. Stupp, Enhanced out-of-plane conductivity and photovoltaic performance in n = 1 layered perovskites through organic cation design. *J. Am. Chem. Soc.* **140**, 7313–7323 (2018).

24. G. Liu, X.-X. Xu, S. Xu, L. Zhang, H. Xu, L. Zhu, X. Zhang, H. Zheng, X. Pan, Passivation effect of halogenated benzylammonium as a second spacer cation for improved photovoltaic performance of quasi-2D perovskite solar cells. *J. Mater. Chem. A* **8**, 5900–5906 (2020).

25. J. Hu, I. W. H. Oswald, S. J. Stuard, M. M. Nahid, N. Zhou, O. F. Williams, Z. Guo, L. Yan, H. Hu, Z. Chen, X. Xiao, Y. Lin, Z. Yang, J. Huang, A. M. Moran, H. Ade, J. R. Neilson, W. You, Synthetic control over orientational degeneracy of spacer cations enhances solar cell efficiency in two-dimensional perovskites. *Nat. Commun.* **10**, 1276 (2019).

26. D. Kim, H. J. Jung, I. J. Park, B. W. Larson, S. P. Dunfield, C. Xiao, J. Kim, J. Tong, P. Boonmongkolras, S. G. Ji, F. Zhang, S. R. Pae, M. Kim, S. B. Kang, V. Dravid, J. J. Berry, J. Y. Kim, K. Zhu, D. H. Kim, B. Shin, Efficient, stable silicon tandem cells enabled by anion-engineered wide-bandgap perovskites. *Science* **368**, 155–160 (2020).

27. Y. Gao, Z. Wei, P. Yoo, E. Shi, M. Zeller, C. Zhu, P. Liao, L. Dou, Highly stable lead-free perovskite field-effect transistors incorporating linear π-conjugated organic ligands. *J. Am. Chem. Soc.* **141**, 15577–15585 (2019).

28. A. H. Proppe, R. Quintero-Bermudez, H. Tan, O. Voznyy, S. O. Kelley, E. H. Sargent, Synthetic control over quantum well width distribution and carrier migration in low-dimensional perovskite photovoltaics. *J. Am. Chem. Soc.* **140**, 2890–2896 (2018).

29. M. Daboczi, S. R. Ratnasingham, L. Mohan, C. Pu, I. Hamilton, Y.-C. Chin, M. A. Mclachlan, J.-S. Kim, Optimal interfacial band bending achieved by fine energy level tuning in mixed-halide perovskite solar cells. *ACS Energy Lett.* **6**, 3970–3981 (2021).

30. Y. Zhao, T. Heumueller, J. Zhang, J. Luo, O. Kasian, S. Langner, C. Kupfer, B. Liu, Y. Zhong, J. Elia, A. Osvet, J. Wu, C. Liu, Z. Wan, C. Jia, N. Li, J. Hauch, C. J. Brabec, A

bilayer conducting polymer structure for planar perovskite solar cells with over 1,400 hours operational stability at elevated temperatures. *Nat. Energy* **7**, 144–152 (2022).

31. Y. Wang, L. Duan, Z. Hameiri, M. Zhang, X. Liu, Y. Bai, X. Hao, PTAA as efficient hole transport materials in perovskite solar cells: A review. *Sol. RRL* **6**, 2200234 (2022).

32. F. M. Rombach, S. A. Haque, T. J. Macdonald, Lessons learned from spiro-OMeTAD and PTAA in perovskite solar cells. *Energ. Environ. Sci.* **14**, 5161–5190 (2021).

33. W. S. Yang, B. W. Park, E. H. Jung, N. J. Jeon, Y. C. Kim, D. U. Lee, S. S. Shin, J. Seo, E. K. Kim, J. H. Noh, S. I. Seok, Iodide management in formamidinium-lead-halide-based perovskite layers for efficient solar cells. *Science* **356**, 1376–1379 (2017).

34. M. Jeong, I. W. Choi, E. M. Go, Y. Cho, M. Kim, B. Lee, S. Jeong, Y. Jo, H. W. Choi, J. Lee, J.-H. Bae, S. K. Kwak, D. S. Kim, C. Yang, Stable perovskite solar cells with efficiency exceeding 24.8% and 0.3-V voltage loss. *Science* **369**, 1615–1620 (2020).

35. H. Ren, S. Yu, L. Chao, Y. Xia, Y. Sun, S. Zuo, F. Li, T. Niu, Y. Yang, H. Ju, B. Li, H. Du, X. Gao, J. Zhang, J. Wang, L. Zhang, Y. Chen, W. Huang, Efficient and stable Ruddlesden–Popper perovskite solar cell with tailored interlayer molecular interaction. *Nat. Photonics* **14**, 154–163 (2020).

36. Z. Liu, L. Qiu, L. K. Ono, S. He, Z. Hu, M. Jiang, G. Tong, Z. Wu, Y. Jiang, D.-Y. Son, Y. Dang, S. Kazaoui, Y. Qi, A holistic approach to interface stabilization for efficient perovskite solar modules with over 2,000-hour operational stability. *Nat. Energy* **5**, 596–604 (2020).

37. M. Stolterfoht, C. M. Wolff, J. A. Márquez, S. Zhang, C. J. Hages, D. Rothhardt, S. Albrecht, P. L. Burn, P. Meredith, T. Unold, D. Neher, Visualization and suppression of interfacial recombination for high-efficiency large-area pin perovskite solar cells. *Nat. Energy* **3**, 847–854 (2018).

38. J. Wang, W. Fu, S. Jariwala, I. Sinha, A. K.-Y. Jen, D. S. Ginger, Reducing surface recombination velocities at the electrical contacts will improve perovskite photovoltaics. *ACS Energy Lett.* **4**, 222–227 (2019).

39. Z. Ni, H. Jiao, C. Fei, H. Gu, S. Xu, Z. Yu, G. Yang, Y. Deng, Q. Jiang, Y. Liu, Y. Yan, J. Huang, Evolution of defects during the degradation of metal halide perovskite solar cells under reverse bias and illumination. *Nat. Energy* **7**, 65–73 (2022).

40. L. J. Farrugia, WinGX and ORTEP for Windows: An update. *J. Appl. Cryst.* **45**, 849–854 (2012).

41. C. B. Hübschle, G. M. Sheldrick, B. Dittrich, ShelXle: A Qt graphical user interface for SHELXL. *J. Appl. Cryst.* **44**, 1281–1284 (2011).

42. G. M. Sheldrick, Crystal structure refinement with SHELXL. *Acta Crystallogr. C* **71**, 3–8 (2015).

43. G. M. Sheldrick, SHELXT–integrated space-group and crystal-structure determination. *Acta Crystallogr. Sect. A* **71**, 3–8 (2015).
